# The sugar transporter STP1-driven trophic conversion of *Chlamydomonas reinhardtii*

**DOI:** 10.1186/s12934-026-02957-4

**Published:** 2026-02-20

**Authors:** Bum-Soo Shin, Yong Jae Lee, Jin-Ho Yun, Dong-Yun Choi, Dae-Hyun Cho, Dong Won Lee, Ji Won Kim, Junyoung Chun, Hong Il Choi, Hee-Sik Kim

**Affiliations:** 1 Cell Factory Research Center, Korea Research Institute of Bioscience and Biotechnology (KRIBB), Daejeon, 34141 Republic of Korea; 2 Department of Environmental Biotechnology, KRIBB School, University of Science and Technology (UST), Daejeon, 34113 Republic of Korea

**Keywords:** Microalgae, *Chlamydomonas reinhardtii*, Glucose transporters, Trophic conversion, STP1, Heterotrophic cultivation

## Abstract

**Background:**

*Chlamydomonas reinhardtii* is a versatile model microalga with strong potential for industrial applications in light of its rapid growth, ability to synthesize valuable metabolites, and GRAS status. However, large-scale phototrophic cultivation faces challenges such as self-shading, uneven illumination, as well as the prerequisite of lighting equipment, all of which limit productivity and economic feasibility. Glucose-based heterotrophic cultivation is a scalable alternative, yet *C. reinhardtii* lacks endogenous glucose transporters, which inherently precludes its glucose uptake.

**Results:**

To overcome this limitation and expand its trophic flexibility, we engineered *C. reinhardtii* by introducing the STP1 glucose transporter originating from *Arabidopsis thaliana*, which confers the ability to uptake glucose. Along with the expression of *STP1* in *C. reinhardtii*, two previously studied glucose transporters were also expressed individually for comparison: *GLUT1* from *Homo sapiens* and *HUP1* from *Parachlorella kessleri* because both are known to enable glucose uptake in *C. reinhardtii*. Resultingly, all transporter-expressing mutants acquired the ability to uptake glucose. Among tested, the *STP1*-expressing strain (3S7) exhibited the highest growth under glucose-supplemented heterotrophic conditions despite the marginal difference. The cell number of the 3S7 strain was 3.56 times higher than that of the wild-type (WT) strain, while the cell numbers of the *GLUT1*- and *HUP1*-expressing strains were 2.84 and 2.79 times higher, respectively. Dry cell mass analysis confirmed the glucose-based heterotrophic growth of the *STP1*-expressing strain, showing a 2.03-fold increase compared to the WT, whose weight is the highest among the transformants. Assays using the fluorescent glucose analog 2-NBDG and the non-metabolizable glucose analog 2-DG validated glucose transport into the 3S7 strain and others. The reduced residual glucose in the culture supernatant also supported the 3S7 strain’s primary ability to uptake glucose compared to the other strains, while exhibiting the biomass yield on glucose of 18.9 ± 8.6%.

**Conclusions:**

These results demonstrate that STP1 can be exploited as a promising glucose importer, conferring glucose uptake ability to *C. reinhardtii*. This study establishes a foundation for increasing its trophic flexibility by broadening its usable organic carbon source, opening up a new opportunity to use the model alga as a cell factory platform for scalable industrial bioprocesses.

**Supplementary Information:**

The online version contains supplementary material available at 10.1186/s12934-026-02957-4.

## Introduction

*Chlamydomonas reinhardtii* is a unicellular green alga with a well-characterized genome and diverse molecular tools, emerging as a prominent model organism in microalgae research [1–3]. Its rapid growth, ability to produce high-value metabolites such as lipids [4], carotenoids [5], and pigments [6], and generally recognized as safe (GRAS) status together underscore the strong potential of *C. reinhardtii* in industrial applications [7]. Among the various cultivation strategies for industrial use, *C. reinhardtii* can be grown under photoautotrophic, mixotrophic, or heterotrophic conditions. However, light-dependent systems such as photoautotrophy and mixotrophy often suffer from low biomass productivity and several scale-up limitations (e.g., self-shading) [8, 9] because of ununiformed illumination, mandating the need for additional lighting equipment that may adversely affect the overall economic competitiveness. In contrast, heterotrophic cultivation under dark conditions bypasses these light-related constraints, enables the use of existing, well-developed industrial fermentation infrastructure. It was reported from previous studies that *C. reinhardtii* has achieved biomass yields up to 40 g L^−1^ under heterotrophic conditions [10], while photoautotrophic and mixotrophic conditions have yielded comparatively lower values up to 1.26 g L^−1^ and 23.69 g L^−1^, respectively, in laboratories [11, 12]. These findings also support that heterotrophic cultivation offers an opportunity to unlock the industrial potential of *C. reinhardtii*.

In heterotrophic cultivation processes of microalgae, the selection of organic carbon sources and their efficient utilization in cellular metabolism directly impacts the processes’ performance. Various organic carbon substrates such as acetate—typically used in hetero- and mixotrophic laboratory cultivations for *C. reinhardtii*, glycerol, lactate, and glucose have been utilized in microalgae cultivation. Among these, glucose is the most universally used substrate for industrial cultivation due to its affordability, chemical stability, and versatile metabolic usage over other sources, all of which make its use inevitable for establishing a large-scale and profitable bioprocess [13]. Yet, *C. reinhardtii* lacks plasma membrane glucose transporters and cannot import glucose into the cytoplasm, posing a primary and major obstacle to glucose-based heterotrophic cultivation. To address this issue, several attempts have been made to introduce exogenous glucose transporters in *C. reinhardtii*. Expression of the human glucose uniporter GLUT1 (glucose transporter 1; *Hs*GLUT1) was found to confer the glucose uptake ability, which led to increase in the cell density under dark conditions [14]. When the hexose-proton symporter, HUP1 (hexose uptake protein 1; *Pk*HUP1), from microalga *Parachlorella kessleri* (formerly known as *Chlorella kessleri*) was expressed, glucose uptake ability was successfully conferred and the cell density increased approximately 1.5-fold compared to the initial inoculum [15]. Although the resulting growth improvement remains unsatisfactory, these results showed that the expression of exogenous glucose importers can mediate glucose-based trophic shift in *C. reinhardtii* and provided future research participant with pivotal options to implement glucose-utilizing *C. reinhardtii* strains (Table 1).

**Table 1:** List of works conferring to glucose uptake ability to *C. reinhardtii*

Inserted genes	Light conditions	Glucose uptake verification methods^*^	References
*HsGLUT1*	In the absence (heterotrophic) and presence (mixotrophic) of light	2-NBDG assay, cell growth measure	[14]
*PkHUP1*	In the absence (heterotrophic) and presence (mixotrophic) of light	d-[U-14 C]-glucose tracing, 2-DG assay, cell growth measure	[15]
*HsGLUT1*, *AtSTP1*, *PkHUP1*	In the absence (heterotrophic) and presence (mixotrophic) of light	2-DG assay, 2-NBDG assay, cell growth measure, HPLC glucose concentration measurement	This study

^*^2-DG and 2-NBDG stand for 2-deoxyglucose and 2-deoxy-2-[(7-nitro-2,1,3-benzoxadiazol-4-yl)amino]-d-glucose, respectively, both of which are glucose analogs

In this study, we then aimed at finding a novel glucose transporter for conferring the glucose uptake ability to *C. reinhardtii* and discovered that the Sugar Transport Protein 1 (STP1) from *Arabidopsis thaliana*—a hexose-proton symporter—can be a promising glucose transporter candidate for the trophic conversion. *At*STP1 is known to play a crucial role in reabsorption of photosynthesized monosaccharides leaked into the extracellular space during the source-sink carbon partitioning, while predominantly located in the transmembrane regions of leaf and stem cells [16–18]. In addition to its corroborated glucose transport role in the photosynthetic organism, several aspects attracted our interest. First of all, its heterologous functional expression was experimentally confirmed in the yeast *Schizosaccharomyces pombe*, implying its wide applicability without host specificity [19]. This study also showed *At*STP1 possesses a high affinity to glucose by demonstrating that its expression decreased the *K*_m_ value to ca. 100 folds, which is up to 20 µM from an intrinsic 3 mM of the wild-type. This property potentially allows for flexibility in substrate concentration for transformants.

In accordance with our expectation, we found that a glucose uptake ability was successfully conferred to a *C. reinhardtii* variant expressing *AtSTP1* (strain 3S7), as verified by multi-layered methods, including the fluorescence and toxicity tests using glucose analogs, and the glucose consumption assay (Table 1). We also found that the *AtSTP1* expression resulted in the heterotrophic growth of the transformant under dark conditions. Furthermore, compared to the control groups, including WT, *HsGLUT1*-, and *PkHUP1*-expressing transformants [14, 15], the 3S7 strain demonstrated the highest heterotrophic productivity and biomass yield on substrate, albeit slightly. Taken together, the strategy of expressing *AtSTP1* heterologously in *C. reinhardtii* could provide a propitious starting point for implementing a complete heterotroph originating from *C. reinhardtii*.

## Materials and methods

### Algal strains culture conditions

**Fig. 1:**
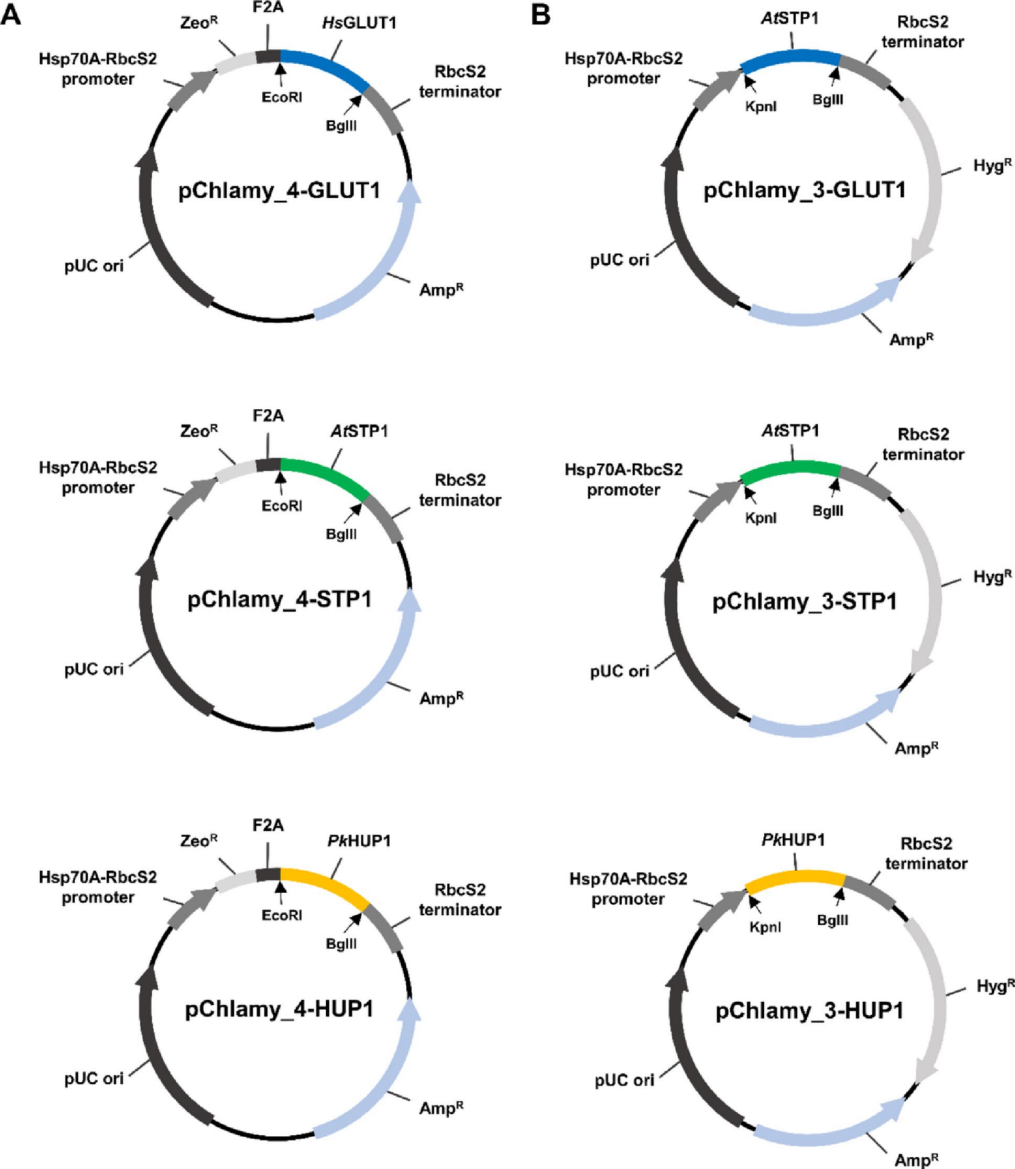
Vector maps of recombinant plasmids, which illustrates the key elements. **A** Vector maps of pChlamy_4-based plasmids harboring *HsGLUT1*, *AtSTP1*, and *PkHUP1*, respectively. **B** Vector maps of pChlamy_3-based plasmids harboring the same set of transgenes

The wild-type (WT) strain *C. reinhardtii* CC125 was obtained from the Chlamydomonas Resource Center at the University of Minnesota (USA). For the evaluation of microalgal growth and seed culture, 8 types of media were used: (1) Tris-acetate-phosphate (TAP) medium [20]; (2) Tris-phosphate medium (TP), which is prepared by excluding acetate from TAP medium and titrated to pH 7.0 with HCl; (3) TP medium containing 5 mM of glucose (TPG-5); (4) TP medium containing 10 mM of glucose (TPG-10); (5) TP medium containing 50 mM of glucose (TPG-50); (6) TP medium containing 100 mM of glucose (TPG-100); (7) TP medium containing 200 mM of glucose (TPG-200); (8) TAP medium containing 10 ppm of hygromycin B; and (9) TAP medium containing 5 ppm of zeocin. In addition, two culture conditions were used for the growth test and seed culture: (1) Light conditions: 25 °C, light intensity of 100 ± 20 µmol m^−2^ s^−1^, 130 rpm; (2) Dark conditions: 25 °C, complete dark conditions, 130 rpm. The WT strain was continuously maintained in TAP medium. Strains harboring the pChlamy_4 vector (Fig. 1A) were cultivated in TAP medium containing zeocin, and those containing pChlamy_3 vector (Fig. 1B) were maintained in TAP medium containing hygromycin B. All strains were initially seeded and cultivated under light conditions.

### In silico structural prediction of glucose transporters

 In silico analyses were performed to characterize the structural features and subcellular localization of three glucose transporters: *At*STP1, *Hs*GLUT1 [21], and *Pk*HUP1 [22]. For in silico analyses, the coding sequences of glucose transporters were retrieved from publicly available databases. The coding sequence of *HsGLUT1* was obtained from the human reference sequence registered under NCBI accession NM_006516, and the coding region of *PkHUP1* was referenced from *HUP1* gene of *Parachlorella kessleri* reported under GenBank accession X55349.1. The sequence of *AtSTP1* was acquired from the *Arabidopsis* Information Resource (TAIR) database using the identifier AT1G11260. To predict the transmembrane topology of each protein, TMHMM v2.0 was used [23]. For identifying their subcellular localizations, DeepLoc v2.1 [24], a deep learning-based predictor for eukaryotic proteins, was employed. The amino acid sequence of each transporter was applied to the publicly available tools to obtain the predictive information on the membrane proteins.

### Plasmid construction and cloning

For heterologous expression in *C. reinhardtii*, commercial expression vectors, the pChlamy_3 and pChlamy_4 vectors (Thermo Fisher Scientific), were used. Both plasmids harbor the constitutive, strong *Hsp70A-RbcS2* fusion promoter and *RbcS2* terminator to drive exogenous gene expression. More specifically, the pChlamy_3 vector carries a hygromycin B resistance gene under the control of the *β2-tubulin* promoter and terminator, while the pChlamy_4 vector carries a zeocin resistance gene and includes a food-and-mouth disease virus 2A (F2A) self-cleaving peptide sequence, allowing bicistronic expression [25]. Based on the reference sequences obtained from public databases, each transporter gene was codon-optimized (Supplementary Information, Table S1) for *C. reinhardtii* using Codon Optimization Tool (GenSmart™ Codon Optimization Tool, https://www.genscript.com/gensmart-free-gene-codon-optimization.html) and synthesized (Bioneer). When the pChlamy_3 vector was used as backbone, the *Kpn*I (Thermo Fisher Scientific) and *Bgl*II (Thermo Fisher Scientific) restriction sites were double-digested, while the *EcoR*I (Thermo Fisher Scientific) and *Bgl*II sites were digested simultaneously when the pChlamy_4 vector was used as backbone. All restriction enzymes were purchased from Thermo Fisher Scientific. After digestion, ligation was conducted using T4 DNA ligase (Promega). 20 µL reaction mixture containing the vector and insert at a 1:3 molar ratio, 1× T4 DNA ligase buffer, and 1 Weiss unit of T4 DNA ligase was incubated at room temperature for 3 h. As a result, 6 recombinant plasmids were constructed (Fig. 1A, B) and the plasmids were directly used for transformation into *Escherichia coli* DH5α competent cells (RBC Bioscience). Plasmid extraction was performed using the Qiagen^®^ Plasmid Maxi Kit (Qiagen).

### Transformation of *C. reinhardtii* and screening of mutant strains

The pChlamy_4-GLUT1, pChlamy_4-HUP1, pChlamy_3-GLUT1, pChlamy_3-HUP1 vectors were linearized by *Sca*I (Thermo Fisher Scientific) restriction enzyme and the pChlamy_4-STP1, pChlamy_3-STP1 vectors were linearized by *Ssp*I (Thermo Fisher Scientific), since the codon-optimized *AtSTP1* sequence contains the *Sca*I restriction site internally. GeneArt^®^ Max efficiency^®^ Transformation Reagent for Algae (Thermo Fisher Scientific) was used as a cell wall permeabilization buffer, and electroporation was performed with a voltage of 500 V, a capacity of 50 µF, and a resistance of 800 Ω for 7.5 × 10^7^ cells mL^−1^ with 4 µg of linearized vector [26]. After electroporation, cells were recovered in 10 mL of TAP medium containing 40 mM of sucrose solution for 14 h with dark conditions. After recovery, transformed cells were plated onto TAP agar plates (agarized with 1.5% plant agar, Duchefa Biochemie) containing an appropriate selection antibiotic: hygromycin B for transformants harboring pChlamy_3 vectors and zeocin for those harboring pChlamy_4 vectors, corresponding to each plasmid’s resistance gene. Plated cells were incubated in smear light conditions until the colonies appeared.

In order to isolate glucose-utilizing candidates from the constructed libraries, an initial growth screening was performed using 12-well plate. Based on previously reported screening strategies for heterotrophic or mixotrophic growth in microalgae, 5 mM of glucose was applied as the screening concentration [14, 15, 27, 28]. Single colonies were individually picked from selective media and pre-incubated in a 96-well plate containing TAP medium supplemented with an appropriate antibiotic. All strains were then seeded in 12-well plate with 1 mL of TPG-5 medium under light and dark conditions for 3 days, respectively. Optical density of cells was measured at 800 nm (OD_800_) using a Spark^®^ Multimode Microplate Reader (Tecan).

After initial screening of the candidate strains, two top-performing strains were selected from each transformant group based on their growth. These candidates’ growth performances were further evaluated in the medium comprising different nutrient combinations, including yeast extract and peptone (YP) and with or without glucose, using a 24-well plate. The cells were incubated for 3 days under light conditions. Used media were prepared based on TP medium containing 3 g L^−1^ of yeast extract and 5 g L^−1^ of peptone, in the presence and absence of glucose (5 mM). OD₈₀₀ was measured on day 0 and 3 using a Spark^®^ Multimode Microplate Reader (Tecan). The relative growth was calculated as the fold change in OD₈₀₀ by dividing the day 3 value by the day 0 value.

Crude PCR was performed after the 12-well plate-based cell growth evaluation. PCR templates were prepared according to the previous study [29]. PCR was performed using KOD Plus Neo polymerase (Toyobo) with an appropriate primer set (Table 2). The reaction conditions were as follows: pre-denaturation at 94 °C for 2 min, denaturation at 98 °C for 10 s, and extension at 68 °C for 1 min. Denaturation and extension steps were repeated for 35 cycles. For each candidate, the respective vectors used for gene insertion were employed as positive controls. The actin gene (*IDA5*; Cre13.g603700) was amplified as a housekeeping gene.

**Table 2:** Primer sets used in this study

Purpose	Primer name	Nucleotide sequence (5ʹ to 3ʹ)
Colony PCR of *HsGLUT1*	F_GLUT1_colP	AGCCCGTGTACGCTACCA
	R_pChlamy_colP	ATTCGCGCTTCAAATACGCC
Colony PCR of *AtSTP1*	F_STP1_colP	AGCCTGATGAGCGCTGT
	R_pChlamy_colP	ATTCGCGCTTCAAATACGCC
Colony PCR of *PkHUP1*	F_HUP1_colP	CACCGGCATCAACGCCAT
	R_pChlamy_colP	ATTCGCGCTTCAAATACGCC
Colony PCR of *Actin*	F_Actin_CTL	TTGGGTAAAGAGGGCGGGAG
	R_Actin_CTL	ACACAGTCCCCATGACCTCCT
qPCR of *HsGLUT1*	F_GLUT1_qPCR	GATCAACGCTCCCCAGAAGG
	R_GLUT1_qPCR	CGCTGAAAATAGCCACGGAC
qPCR of *AtSTP1*	F_STP1_qPCR	GAGCATTACCGTGTCCGTCA
	R_STP1_qPCR	TCGACATGACCACCACGAAG
qPCR of *PkHUP1*	F_HUP1_qPCR	CTCGACCCTGATTGCTGTG
	R_HUP1_qPCR	AGCGAACTCGATAGCCAGG
qPCR of *RACK1*	F_RACK1_qPCR	AGATCTGGGACCTGGAGAGCAAG
	R_RACK1_qPCR	TGGGCATTTACAGGGAGTGGC

### Transcriptional and protein expression analyses

Total RNA was extracted from all strains using the Total RNA Purification Kit for Plants (Nanohelix), and cDNA was synthesized with the GoScript™ Reverse Transcription System (Promega) under the following conditions: 25 °C for 5 min, 42 °C for 1 h, and 70 °C for 15 min. Transcription levels of each gene were quantified using iQ™ SYBR^®^ green Supermix (Bio-Rad) with an appropriate primer set (Table 2). Amplification reactions were conducted on a CFX Connect Real-Time PCR Detection System (Bio-Rad). qPCR was performed under the thermal cycling conditions: polymerase activation and cDNA pre-denaturation at 95 °C for 3 min; denaturation at 95 °C for 15 s; annealing and extension at 55 °C for 1 min; and data acquisition step; which was repeated for 40 cycles. The expression level of the Receptor for Activated C Kinase 1 (*RACK1*; Cre06.278222) gene was estimated as an endogenous control. The relative expression levels were calculated using the 2^−ΔCt^ method.

To assess the protein expression levels of glucose transporters, *Hs*GLUT1 protein was selected as a representative target. For protein extraction, 10 mL of 3G16 cells were sampled when the OD_800_ reached 0.4. After centrifuge at 4000 ×*g* for 10 min, cell pellets were resuspended in 4× Laemmli Sample Buffer (Bio-Rad) containing 50 mM of dithiothreitol (DTT). The sample mixture was then heated at 70 °C for 10 min, followed by centrifugation at 20,000 ×g for 5 min. Supernatants were collected as protein samples for western blotting [30]. Protein concentrations were determined using the Pierce™ BCA Protein Assay Kit (Thermo Fisher Scientific). Each sample was diluted to contain 200 µg of total protein in a final volume of 40 µL and loaded onto a Mini-PROTEIN TGX Stain-Free Gel (Bio-Rad). Sodium dodecyl sulfate (SDS)–polyacrylamide gel electrophoresis (SDS-PAGE) was then performed with a voltage of 40 V at 4 °C overnight using Tris-glycine-SDS buffer (LPS Solution). Precision Plus Protein Dual Color Standards (Bio-Rad) was used as a ladder for estimating the molecular weight of proteins. Subsequently, the loaded proteins were transferred to a polyvinylidene fluoride (PVDF) membrane with a voltage of 40 V at 4 °C for 8 h. Following protein transfer, the membrane was blocked overnight at 4 °C with Tris-buffered saline containing 5% Tween-20 (TBST; LPS solution), supplemented with 5% (w/v) skim milk (Bio-Rad). The membrane was then incubated with an anti-GLUT1 Antibody (Sigma-Aldrich) diluted to 1:3000 in TBST containing 5% (w/v) skim milk overnight at 4 °C. After incubation, the membrane was washed with TBST for 15 min, followed by three additional washings of 5 min each. The membrane was then incubated with the horse radish peroxidase (HRP)-conjugated anti-rabbit IgG secondary antibody (Abcam) diluted to 1:3000 in TBST containing 5% (w/v) skim milk overnight at 4 °C. The membrane was rewashed in the same manner. Protein bands were visualized using the ChemiDoc™ Imaging System (Bio-Rad), through the reaction between HRP-conjugated secondary antibody and enhanced chemiluminescence (Femto; Thermo Fisher Scientific). For the detection of housekeeping protein, the expression of the α-tubulin protein was analyzed. Following the detection of GLUT1, antibodies were stripped using a glycine-based stripping buffer (25 mM glycine, 1% SDS, pH adjusted to 2.0 with HCl). The membrane was then incubated with the anti-α-tubulin primary antibody (1:5000; Abcam) in TBST containing 5% (w/v) skim milk overnight at 4 °C, followed by incubation with the HRP-conjugated anti-mouse IgG secondary antibody (1:10,000; Abcam) using the same washing and detection conditions described above.

### Assessment of glucose uptake using glucose analog-based assay

2-Deoxy-2-[(7-nitro-2,1,3-benzoxadiazol-4-yl)amino]-d-glucose (2-NBDG) is a fluorescent glucose analog used to assess cellular glucose uptake ability [31]. Fluorescence levels of all transformants were measured using the Glucose Uptake Cell-Based Assay Kit (Cayman Chemical). 1 mL of cultivated cells were harvested in the early logarithmic phase, washed, and resuspended in 1 mL of TP medium. 10 µL of 10 mg mL^−1^ 2-NBDG solution was added, and the cells were incubated in dark conditions for 12 h. After incubation, cells were washed twice with 400 µL of Cell-Based Assay Buffer, resuspended with 100 µL of the same buffer, and fluorescence levels were measured using Spark^®^ multimode Microplate Reader (Tecan). Optical density was measured at a wavelength of 800 nm and fluorescence levels were measured at an excitation wavelength of 485 nm and an emission wavelength of 535 nm. Prior to data analysis, buffer-only wells were measured as blanks to set the baselines both for optical density and fluorescence. Fluorescence values were also normalized by the corresponding blank-corrected cell density (as the optical density). Outliers in the evaluated data were excluded using the interquartile range (IQR) method.

To detect the fluorescence image, a Zeiss laser scanning confocal microscope (LSM800, Carl Zeiss) was used. 2-NBDG was detected at an excitation wavelength of 488 nm and an emission wavelength of 562 nm, while chlorophyll was detected at an excitation wavelength of 488 nm and an emission wavelength of 688 nm. All images were acquired in 16-bit mode with a digital gain of 1, a digital offset of 0, and a pinhole set to 1.0 Airy unit, using a 10× objective with 1.0× zoom. All strains were imaged under identical acquisition conditions using Zen 2 software (Carl Zeiss). For display, a gamma value of 1.0 was applied, and the dynamic range was standardized with black and white levels set to 0 and 25,000, respectively. These display settings were uniformly applied across all samples to ensure consistent visualization without altering the underlying fluorescence intensity values. Fluorescence quantification was performed using ImageJ v1.54 software (National Institutes of Health) and the normalized fluorescence intensity was calculated by dividing the integrated fluorescence of each region of interest (ROI) by its area.

2-Deoxyglucose (2-DG)-based cell growth inhibition test was also performed to evaluate glucose uptake ability. 2-DG, an analog of glucose, is toxic to the organisms that can uptake and utilize glucose as an energy source [32]. To confirm the glucose utilization ability of transformants, cells were cultivated in TP medium containing 0, 10, 25, and 50 mM of 2-DG, respectively. The initial cell concentration was adjusted to an OD_800_ of 0.15 under the light conditions. The observation lasted for 8 days.

### Quantification of residual glucose in the supernatant by HPLC

To measure the residual glucose in the culture medium after cultivation, all strains were cultivated under dark conditions in TP medium supplemented with 5, 10, and 100 mM of glucose, with the initial cell density adjusted to an OD_800_ of 0.5. Cultivation was maintained in a 250 mL flask with 50 mL (working volume) of medium and incubated for 8 days. To calculate the glucose-to-biomass yield, dry cell weight (DCW) was measured on day 8. For DCW measurement, 10 mL of culture was collected and the cells were washed twice with deionized water to avoid interference from glucose residues. The suspension was then filtrated through a 47 mm glass microfiber filter (Whatman) that had been pre-weighed prior to sample loading. After filtration, the filter was dried overnight at 60 °C and re-weighed to obtain the DCW.

Glucose concentration was measured on days 0, 4, and 8. Supernatant of the cultivated samples was collected and filtrated. To minimize analytical errors during quantification, samples were diluted at different ratios depending on the glucose concentration of the culture medium. Specifically, samples from the TPG-100 conditions were diluted 100-fold, whereas those from the TPG-5 and TPG-10 conditions were diluted 20-fold using LC-grade water. Glucose concentrations were then determined using high-performance liquid chromatography (HPLC; Agilent). The HPLC analysis was performed using an Aminex HPX-87 H column (Bio-Rad), with a column temperature of 50 °C, an injection volume of 10 µL, and a flow rate of 0.6 mL min^−1^. The mobile phase consisted of 5 mM sulfuric acid in LC-grade water and detection was performed using a refractive index (RI) detector. For relative quantification, a standard calibration curve was generated using glucose solutions prepared in LC-grade water at concentrations of 0.25, 0.5, 1, and 2 g L^−1^. Outliers in the evaluated data were excluded using the IQR method.

To evaluate the glucose utilization efficiency of mutant strains, the glucose-to-biomass conversion yield (%)—as biomass yield on substrate—was calculated by dividing the increase in DCW (in g) after 8 days of cultivation by the amount of glucose consumed (in g). The amount of glucose consumed by each mutant strain was calculated by subtracting the residual glucose concentration of mutant from that of the WT, measured under the same conditions on day 8. The increase in DCW was also calculated by subtracting the average DCW of the WT strain from that of each mutant strain on day 8.

### Statistical analysis

All experiments were performed with at least biological or technical replicates as described in figure legends. Except for the heat-map graph, all growth data are presented as the mean ± standard error. Statistical significance was evaluated using a two-tailed Student’s *t*-test using Microsoft Excel software: [*] means the *p*-value is lower than 0.05; [**] the *p*-value is lower than 0.01; and [***] means the *p*-value is lower than 0.001.

## Results and discussion

### Selection of glucose transporter-expressing transformants

Prior to the practical experiments, in silico analyses were performed to predict the topology and localization of the exogenous glucose transporters (i.e., *At*STP1, *Hs*GLUT1, and *Pk*HUP1) for expression in *C. reinhardtii*—a prerequisite for them to be properly functional. TMHMM v2.0, which predicts transmembrane regions based on a hidden Markov model with a high sensitivity and reliability, confirmed that *Hs*GLUT1, *At*STP1, and *Pk*HUP1 possess 12 transmembrane helices (Table 3). This structure is a common feature of glucose transporters across diverse organisms including humans [21], plants [19], microalgae [22], and yeast [33]. Thus, it can be considered a hallmark of membrane transport proteins. Protein localization was also assessed using DeepLoc v2.1, a deep learning-based tool that integrates sequence-based features with neural networks to predict subcellular localization. Similarly, all transporters were predicted to localize to the membrane. These results strongly suggested that *At*STP1, *Hs*GLUT1, and *Pk*HUP1 are proper membrane-localized candidates to confer a glucose uptake ability to *C. reinhardtii* (Table 3 and Supplementary Information, Fig. S1).

**Table 3:** In silico analyses for glucose transporters

Glucose transporters	Topology analysis	Localization analysis
	TMHMM v2.0	DeepLoc v2.1
*AtSTP1*	12 transmembrane helices	Transmembrane
*HsGLUT1*	12 transmembrane helices	Transmembrane
*PkHUP1*	12 transmembrane helices	Transmembrane

Followed by in silico analyses, *AtSTP1*, *HsGLUT1*, and *PkHUP1* were expressed in *C. reinhardtii*, respectively. Among these three transporters, since the function of *Hs*GLUT1 and *Pk*HUP1 have been proved in previous studies (Table 1), these genes were expressed for comparison with the *AtSTP1* expressing strains. First, we attempted to screen glucose-utilizing *C. reinhardtii* strains from transformant libraries in the dark with a relatively low glucose concentration (i.e., 5 mM), considering the possible mutual effects of light and the organic carbon source (Supplementary Information, Fig. S2A) [34]. As a result, no statistically significant differences in growth were observed between the tested candidates and the WT, likely due to insufficient cell viability under this cultivation mode, which may have masked the functional contribution of the introduced glucose transporters. This finding is consistent with a previous report showing that relatively low glucose concentrations are insufficient to induce full heterotrophy in the non-native glucose-utilizing microalga, *Phaeodactylum tricornutum* [27].

We then conducted the primary screening under light conditions to maintain minimal cellular viability at the relatively low glucose concentration in the dark. At this stage, mutants harboring the pChlamy_3-based vectors demonstrated a better growth compared to those carrying the pChlamy_4-based constructs (Supplementary Information, Fig. S2B). Among the pChlamy_3-GLUT1 transformants, the 2nd (hereafter named 3G2) and 16th (hereafter named 3G16) strains (strain numbered in the order of colony picking) exhibited relatively higher growth rates compared to other colonies within the group with statistical significance, with OD_800_ values increasing 59.7% and 46.3% from day 0 to day 3, respectively. For pChlamy_3-STP1 transformants, the 7th (hereafter named 3S7) and 10th (hereafter named 3S10) strains were identified as the most promising candidates based on their statistical growth performance test, corresponding to 34.6% and 29.7% increases in OD_800_ for the same time period, respectively. In case of pChlamy_3-HUP1 transformants, the 6th (hereafter named 3H6) and 18th (hereafter named 3H18) strains showed superior growth with statistical significance, with 70.8% and 67.0% increased OD_800_ during the three days, respectively (Supplementary Information, Fig. S2B).

To further evaluate the performance of these selected strains under nutrient-enriched conditions—for simulating conditions presuming a future industrial fermentation scenario, additional growth test was conducted using TP medium supplemented with YP, in the presence and absence of glucose. As a result, for the 3S7 and 3H6 strains, we found that the glucose supplementation improved their growths under nutrient-enriched conditions with statistical significance, and at the same time, the growth performances were much higher than those of the 3S10 and 3H18 strains statistically, respectively (Supplementary Information, Fig. S3). This suggests the 3S7 and 3H6 strains can be exploited with a wide range of nutrient conditions compared to their respective competitor strains. For the *HsGLUT1*-expressing strains, the 3G16 strain’s growth was increased in the presence of glucose under nutrient-enriched conditions with statistical significance, while the proliferation of the 3G2 strain under the same conditions was rather inhibited compared to that in the absence of glucose, implying that the 3G2 strain possesses low potential as a platform strain to implement a *C. reinhardtii*-based industrial bioprocess (Supplementary Information, Fig. S3). According to the selection criteria, the 3G16, 3S7, and 3H6 strains were selected as the final candidates for upcoming in-depth assessments in this study.

The colony-dependent differences in growth despite having the same vector may be attributed to the random integration nature of the vector in the alga, which causes variation in insertion sites (i.e., positioning effect) that can affect gene expressions and growth outcomes [35]. When the pChlamy_4 vector was used as a backbone, mutant strains exhibited relatively lower growth rates compared to the strains constructed based on the pChlamy_3 vector. This is likely due to low cleavage efficiency of the 2A self-cleaving peptide sequence [36].

### Confirmation of gene insertion and evaluation of transcriptional and translational expression

#### Confirmation of gene integration in transformants

**Fig. 2:**
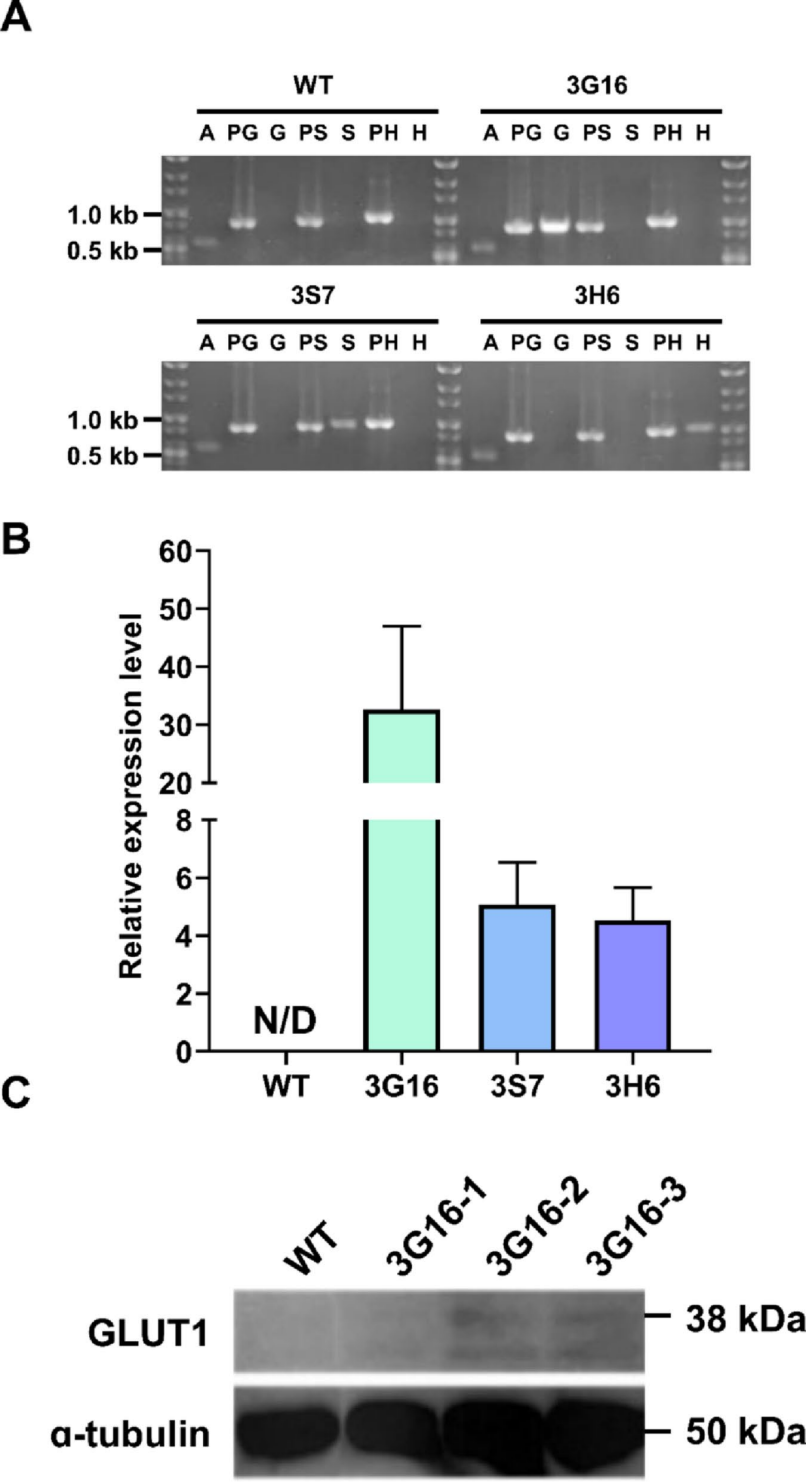
Confirmation of gene integration, transcription, and protein expression in transformants. **A** Colony PCR verification of the experimental strains. Each abbreviation stands for: A: Actin gene (housekeeping gene); PG: positive control of *HsGLUT1*; G: *HsGLUT1*, PS: positive control of *AtSTP1*; S: *AtSTP1*; PH: positive control of *PkHUP1*; H: *PkHUP1*. The positive controls were amplified using plasmid DNA templates of the pChlamy_3-GLUT1, pChlmay_3-STP1, and pChlamy_3-HUP1 vectors, respectively. Genomic DNA from each strain was used as a template to amplify the A, G, S, and H genes. **B** Transcriptional levels of each transporter gene were analyzed by qPCR. The expression levels of *RACK1* were estimated as an endogenous control. Experiments were conducted in technical triplicates (*n* = 3). Error bar represents the standard deviation of the mean. N/D stands for “not determined.” **C** Proper protein expression of *HsGLUT1* was representatively verified by western blotting

To verify successful gene insertion, colony PCR was performed following the growth performance evaluation. Primer sets specifically designed for each glucose transporter gene were applied to confirm the integration of the intended genes in the candidates’ genomic DNA. We then validated that the gene of interest was successfully integrated in the relevant strain (Fig. 2A).

#### Transcription levels analysis of glucose transporter genes

Transcription levels of the glucose transporter genes in 3G16, 3S7, and 3H6 strains were evaluated by qPCR. Each strain was subjected to qPCR analysis using primer sets specific to its respective inserted glucose transporter gene (Table 2). The relative expression levels were normalized to the expression level of the *RACK1* of each transformants. Among the tested strains, the 3G16 exhibited significantly higher expression compared to the 3S7 and 3H6 (Fig. 2B). The transcription level difference was likely due to strain-dependent transcriptional activity or gene integration position effects, which awaits further validations regarding functional glucose uptake ability.

#### Verification of protein expression

To avoid potential issues related to protein misfolding or functional impairment, no protein tags were fused during the construction of expression vectors in this study. While such tags are commonly used to facilitate protein detection, their presence can interfere with the proper folding or activity of transporter proteins [37]. Due to the absence of tags, detection of *At*STP1 and *Pk*HUP1 were unable, as no specific commercial antibodies are currently available. In contrast, since there are commercially available antibodies for *Hs*GLUT1, the expression of *HsGLUT1* at the protein level was representatively assayed using western blotting. Three colonies designated as 3G16-1, 3G16-2, and 3G16-3 were picked from single colonies formed on a selective agar during the sub-cultivation of 3G16. These colonies were individually cultivated and analyzed to account for potential clonal variation in transgene expression.

Western blot analysis of the 3G16 protein sample for *Hs*GLUT1 detection revealed two bands around 37 kDa and 25 kDa—approximately estimated based on the protein standard ladder (Fig. 2C), whose molecular masses were slightly lower than those reported from previous studies. A study on the *HsGLUT1* expression in Chinese hamster ovary cells reported that the native *Hs*GLUT1 exhibits a molecular weight of ca. 50 kDa, whereas a glycosylation-defective mutant, in which a single amino acid was altered to prevent the protein from glycosylation, produced a band at 38 kDa [38]. Meanwhile, in algal systems, such as *C. reinhardtii* and *P. tricornutum*, expression attempts of the *Hs*GLUT1 protein showed a reduced molecular mass of ca. 43 kDa, while displaying two distinctive polypeptide bands in some cases (i.e., 44 and 39 kDa) [14, 39], which was likely attributed to the absence of glycosylation. According to the previous cases, it can be anticipated that the *Hs*GLUT1 protein expression in different host could lead to different molecular weight depending upon its glycosylation status. Therefore, we could reach a conclusion that the GLUT1 protein was successfully expressed in the 3G16 strain with a non-glycosylated state.

### Glucose analog-based analysis of glucose uptake effects

**Fig. 3:**
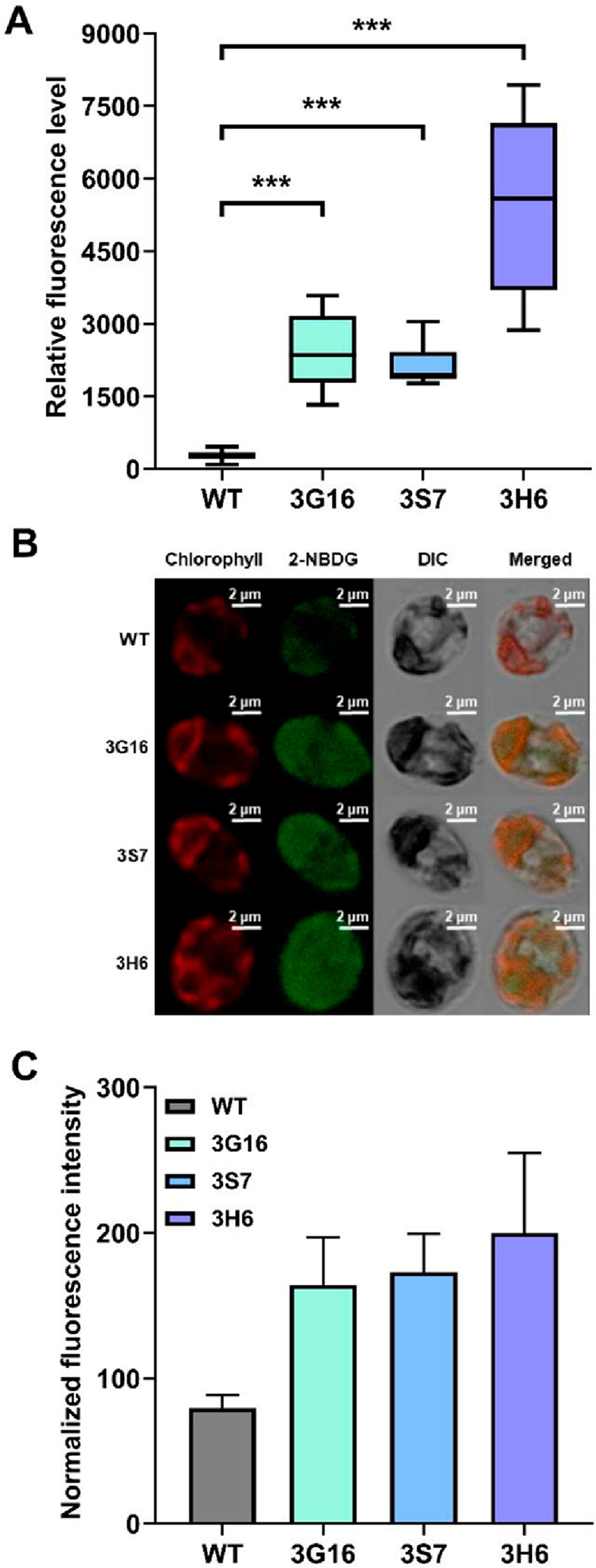
Assessment of glucose uptake through 2-NBDG assay. **A** Box and whisker plots demonstrate relative fluorescence levels of each mutant cell derived from biological quadruplicates with respective technical quadruplicates (*n* = 16 in total). The median (the center) ± whiskers (1.5× the interquartile range from the lower quartile and upper quartile) with the interquartile boundaries are provided. [***] indicates an extremely significant difference (*p* < 0.001) compared to WT. **B** Confocal microscopy images visualize the uptake of 2-NBDG into transformants and its fluorescence expression. DIC stands for differential interference contrast. Merged channel demonstrates the combination of the images from three different channels. **C** Quantification of 2-NBDG uptake presented as normalized fluorescence intensity values of confocal images in **B** and Supplementary Information, Fig. S4. Fluorescence intensity was quantified from three independent cells from each strain (*n* = 3)

To evaluate glucose uptake by the transformants, the fluorescent glucose analog 2-NBDG was used primarily. Compared to the WT, the levels of fluorescence in the 3G16, 3S7, and 3H6 mutants were 8.7-, 7.6-, and 19.1-fold higher, respectively (Fig. 3A). Consistently, increased fluorescence signals were observed in the transformants for the 2-NBDG channel compared to that observed in the WT (Fig. 3B). To further quantify intracellular glucose uptake at the single-cell level, 2-NBDG fluorescence intensities were additionally analyzed by ROI-based image quantification using ImageJ (Fig. 3B and Supplementary Information, Fig. S4). As a result, ROI-normalized fluorescence intensities were found to be higher in all three transformants compared to the WT (Fig. 3C). The average normalized fluorescence intensity values were 79.5 for WT, 164.1 for 3G16, 173.1 for 3S7, and 199.9 for 3H6. While a basal fluorescence signal was detectable in the WT cells, likely reflecting intrinsic cellular background or nonspecific signal, expression of glucose transporters markedly enabled intracellular accumulation of 2-NBDG. Taken together, these results support that heterologous expression of glucose transporters enables effective uptake of the fluorescent glucose analog into *C. reinhardtii* cells.

**Fig. 4:**
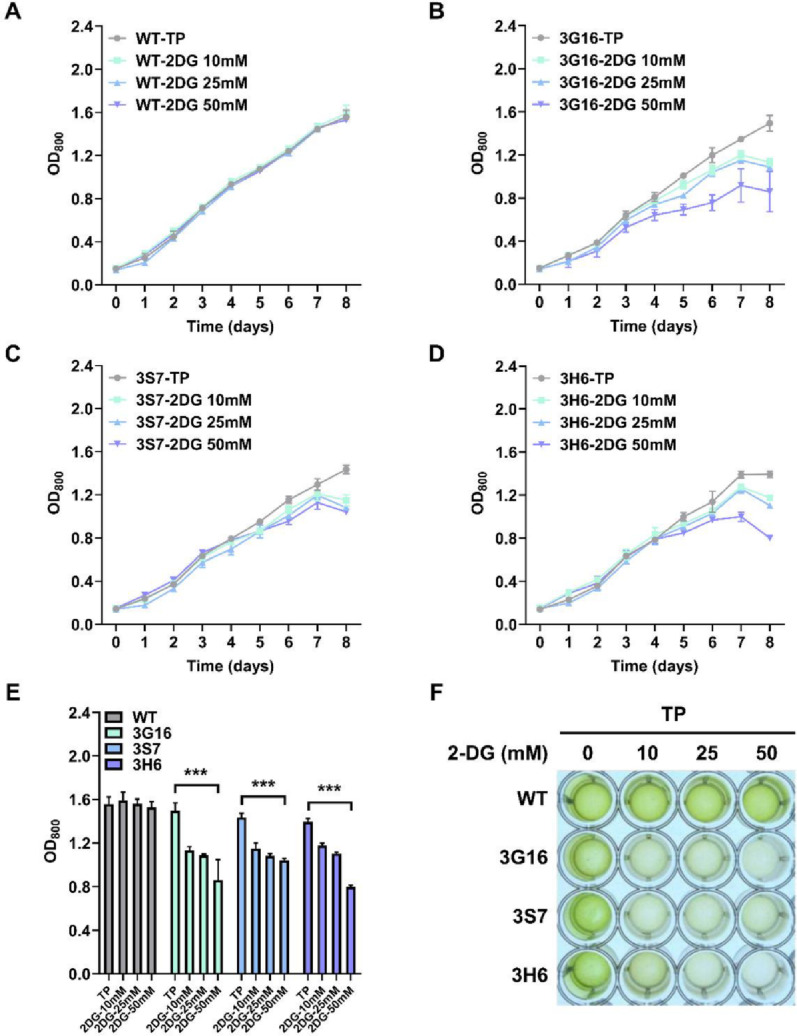
2-DG-based cell growth inhibition test. 2-DG concentration in TP medium were 0, 10, 25, 50 mM, respectively. **A** Growth curves of WT cells based on OD_800_ measurements. **B** Growth curves of 3G16 cells based on OD_800_ measurements. **C** Growth curves of 3S7 cells based on OD_800_ measurements. **D** Growth curves of 3H6 cells based on OD_800_ measurements. **E** Comparison of differences in OD_800_ values on day 8. [***] indicates an extremely significant difference (*p* < 0.001) compared to each cell line cultivated in TP medium. **F** Image of cells on day 8. Experiments were conducted in biological duplicates and technical duplicates (*n* = 4 in total). Error bar represents the standard deviation of the mean

We extended our analysis to another glucose analog, 2-DG. The glucose analog 2-DG can be assimilated by glucose-consuming strains and phosphorylated to 2-DG-6-phosphate. However, it cannot be further isomerized to fructose-6-phosphate in the glycolytic pathway. As a result, it accumulates intracellularly and inhibits the cell growth [32, 40]. Due to this property, 2-DG has been widely employed to assess glucose uptake capacity across diverse organisms. In WT cells, no significant growth inhibition effect was observed in any of the tested media (Fig. 4A). In contrast, all mutant strains exhibited growth inhibition across all concentrations of 2-DG. In strains 3G16, 3S7, and 3H6, no notable differences in growth were observed during the early- and mid-logarithmic phases of cultivation across all 2-DG concentrations in TP medium. However, significant reductions in cell density appeared in the late-logarithmic phase (Fig. 4B–D). When cultivated in TP medium containing 10 mM of 2-DG, cell growth decreased by 24.2%, 20.0%, and 15.6% in 3G16, 3S7, and 3H6, respectively, compared to growth in TP medium. At 25 mM of 2-DG, reductions of 27.3%, 24.7%, and 20.8% were observed in the same strains. At the highest concentration tested, growth was reduced by 42.5%, 27.5%, and 42.5% in each strain (Fig. 4E). On day 8, all mutant strains showed significantly lower cell densities compared to WT (Fig. 4F). The 2-DG growth inhibition test demonstrated that, unlike the WT, all glucose transporter-expressing mutants exhibited growth suppression, indicating active intracellular transport of glucose analogs. Furthermore, the degree of growth inhibition was proportional to the concentration of 2-DG, suggesting a dose-dependent relationship between 2-DG and growth inhibition.

### Growth evaluation under glucose-supplemented conditions

**Fig. 5:**
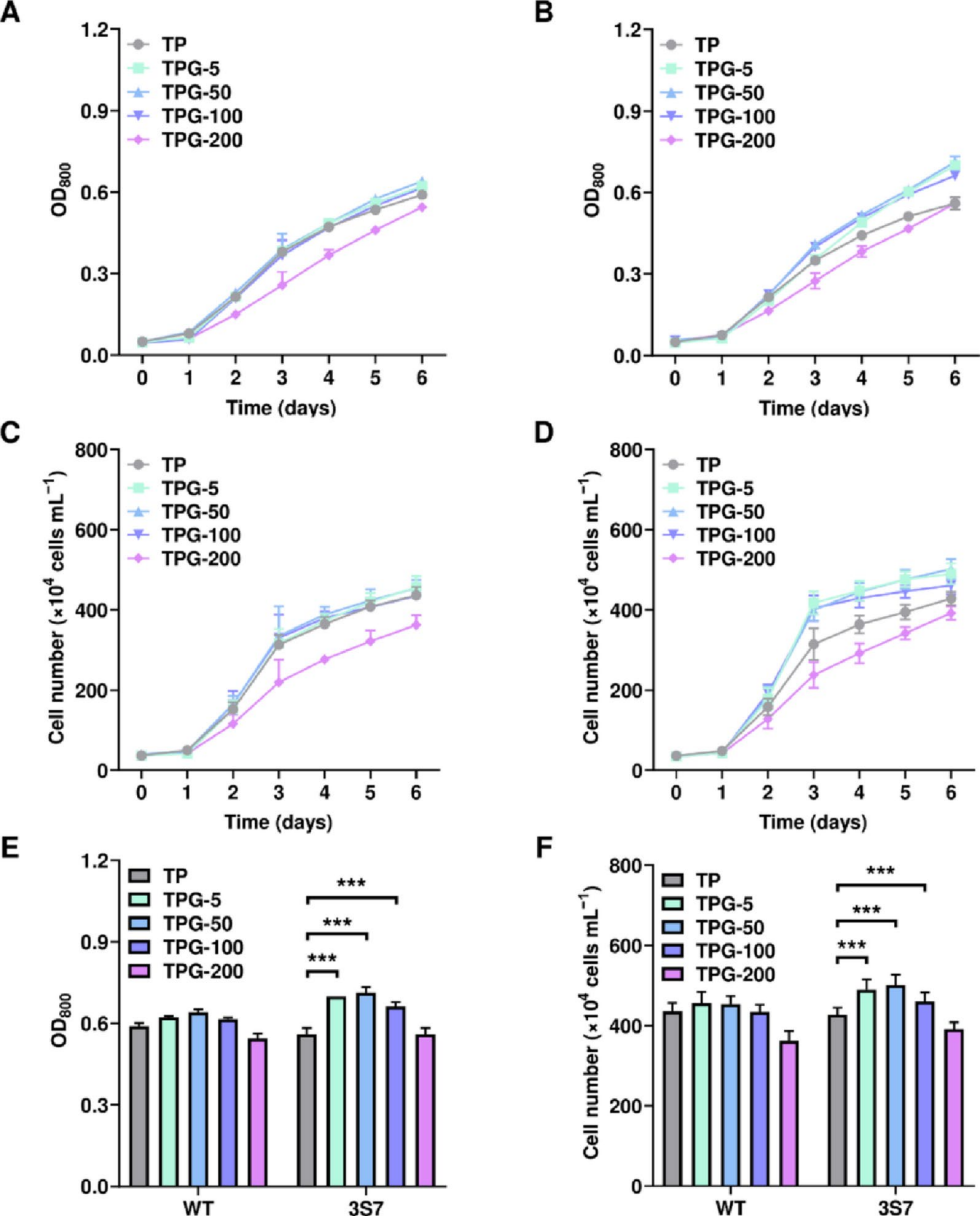
Growth profiles of the 3S7 strain cultivated under light conditions with varying glucose concentrations. TPG-5, TPG-50, TPG-100, and TPG-200 medium were used for the growth test. **A** OD_800_ data of WT. **B** OD_800_ data of 3S7. **C** Cell numbers of WT. **D** Cell numbers of 3S7. **E** OD_800_ data on day 6. **F** Cell numbers data on day 6. OD_800_ measurements were conducted in biological duplicates and technical duplicates (*n* = 4 in total), and cell number measurements were conducted in biological duplicates and technical quadruplicates (*n* = 8 in total). [***] indicates an extremely significant difference (*p* < 0.001) compared to cells cultivated in TP medium in a statistical perspective. Error bar represents the standard deviation of the mean

To determine the optimal glucose concentration for subsequent heterotrophic cultivation, cell growth was evaluated under varying glucose concentrations. The WT strain was used as a control, and the mutant strain 3S7 was selected for in-depth analysis. The media used for evaluation included TP, TPG-5, TPG-50, TPG-100, and TPG-200, and cultivation was conducted under light conditions with an initial OD_800_ of 0.05. This evaluation was designed based on the results of the previous 2-DG experiment, in which growth inhibition was observed up to 50 mM, suggesting that mutant strains’ glucose uptake capacity could be more than 50 mM. Considering this, glucose concentrations were tested up to 200 mM to determine which concentration supported the highest level of growth. After six days of cultivation, no significant differences were observed among the different media conditions for the WT (Fig. 5A, C). However, for the 3S7 strain, all media conditions exhibited higher growth compared to the TP medium (Fig. 5B, D). Specifically, on day 6, the OD_800_ for the 3S7 strain was 0.56, 0.7, 0.715, 0.6625, and 0.56 for TP, TPG-5, TPG-50, TPG-100, and TPG-200 media, respectively (Fig. 5E). Correspondingly, cell numbers were 4.28, 4.90, 5.02, 4.60, and 3.92 × 10^4^ cells mL^−1^, indicating higher growth in all TPG media compared to TP except for the TPG-200 medium (Fig. 5F). Among the tested conditions, the TPG-50 medium presented the highest growth. The 3S7 strain demonstrated a gradient increase in growth with glucose concentrations up to 50 mM, whereas higher concentrations of 100 mM and 200 mM resulted in a decrease in growth. Similarly, the WT strain exhibited a decrease in cell density when cultivated in media containing 200 mM of glucose, which is likely attributed to osmotic stress induced by high glucose concentrations. Based on these results, the TPG-50 medium was selected for subsequent heterotrophic cultivation, as it provided the balance for enhanced growth without the adverse effects of higher glucose concentrations.

**Fig. 6:**
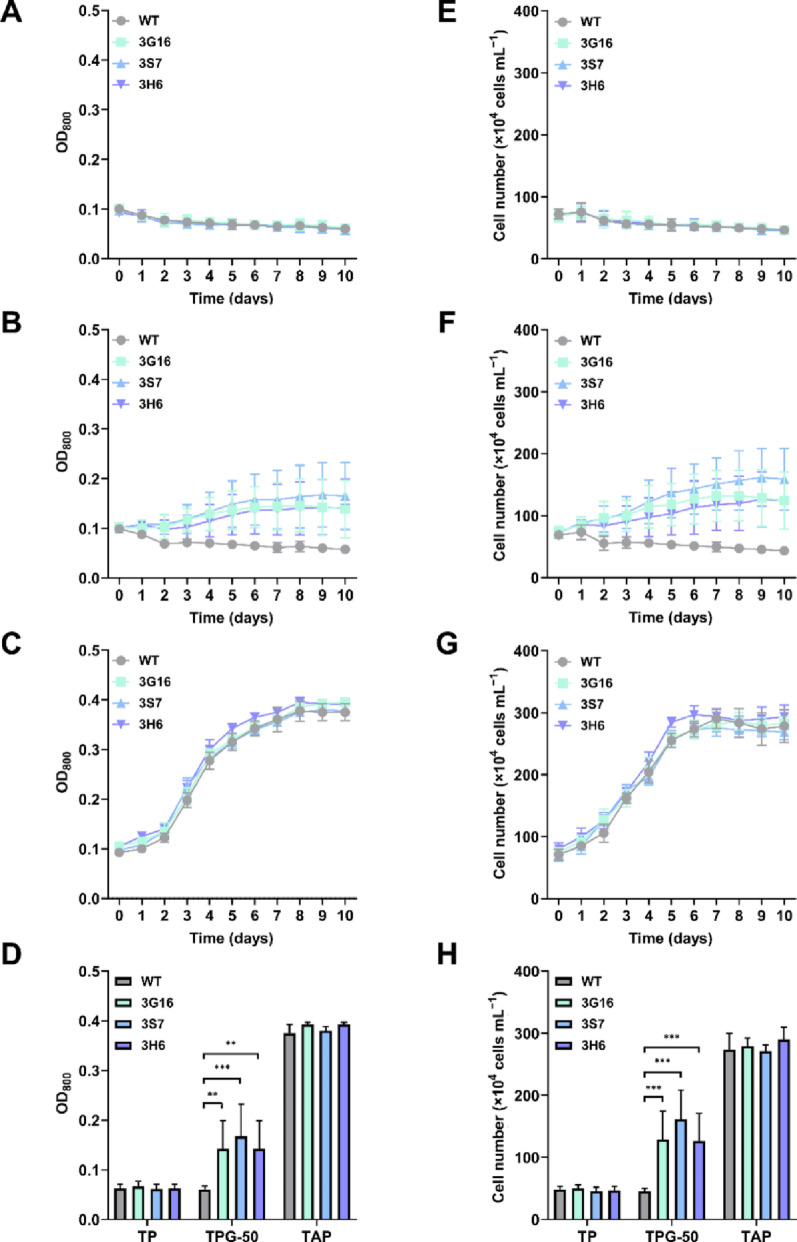
Growth of WT and mutant strains in dark conditions. **A** OD₈₀₀ of cells cultured in TP medium. **B** OD₈₀₀ of cells cultured in TPG-50 medium. **C** OD₈₀₀ of cells cultured in TAP medium. **D** OD₈₀₀ values on day 9. **E** Cell number of cells cultured in TP medium. **F** Cell number of cells cultured in TPG-50 medium. **G** Cell number of cells cultured in TAP medium. **H** Cell number on day 9. For OD_800_ measurements, experiments were conducted using biological quadruplicates with technical duplicates for TP and TPG-50 conditions (*n* = 8 in total), and biological duplicates with technical duplicates for TAP conditions (*n* = 4 in total). For cell number measurements, experiments were conducted using biological quadruplicates with technical quadruplicates for TP and TPG-50 conditions (*n* = 16 in total), and biological duplicates with technical quadruplicates for TAP conditions (*n* = 8 in total). [**] and [***] indicate a highly significant difference (*p* < 0.01) and an extremely significant difference (*p* < 0.001) compared to the WT, respectively. Error bar represents the standard deviation of the mean

Heterotrophic cultivation was then conducted under dark conditions for 10 days in TP, TPG-50, and TAP (as a benchmark organic carbon source for heterotrophy in *C. reinhardtii*) media, respectively. All strains were pre-cultivated in TAP medium for 3 days to reach the early logarithmic phase. After pre-culture, the cells were washed twice with TP medium before conducting the growth evaluation. Because of its low growth rate, the initial OD_800_ was set to 0.1. In TP medium, none of the strains exhibited growth in dark conditions, and the cell numbers were gradually decreased (Fig. 6A, E). In contrast, in TPG-50 medium, the cell numbers of the WT were gradually decreased, while mutant strains expressing glucose transporters cell numbers were increased (Fig. 6B, F). On day 9, OD_800_ were 0.06, 0.1425, 0.1675, and 0.1425 for WT, 3G16, 3S7, and 3H6, respectively, representing 2.38-, 2.79-, and 2.38-fold increases in 3G16, 3S7, and 3H6 compared to the WT (Fig. 6D). Cell numbers on day 9 were 4.55 × 10^5^, 1.29 × 10^6^, 1.62 × 10^6^, and 1.27 × 10^6^ cells mL^−1^, with 3G16, 3S7, and 3H6 showing 2.84-, 3.56-, and 2.79-fold higher than the WT (Fig. 6H). As a benchmark, growth evaluations were also performed in TAP medium under dark conditions. Under these conditions, all strains exhibited comparable growth. On day 9, OD_800_ value were 0.375, 0.3925, 0.38, and 0.3925 for WT, 3G16, 3S7, and 3H6, respectively. Consistently, cell numbers on day 9 were 2.74 × 10^6^, 2.79 × 10^6^, 2.71 × 10^6^, and 3.00 × 10^6^ cells mL^−1^ for WT, 3G16, 3S7, and 3H6, respectively.

The results demonstrate that all engineered strains are capable of sustaining growth in the dark using glucose as the sole carbon source. Among the strains, the 3S7 strain expressing *AtSTP1* demonstrated the most remarkable cell growth compared to other glucose transporter-expressing transformants. Two potential situations for this were considered. First, the superior transport capacity of the *AtSTP1* glucose transporter, resulting in enhanced glucose uptake ability compared to other glucose transporters, and second, the strain’s exceptional ability to utilize glucose efficiently. Nevertheless, compared to the acetate-supported growth – the conventional heterotrophic cultivation condition for *C. reinhardtii*, glucose-supported growth remained unsatisfactory. In terms of cell numbers, the 3S7 strain exhibited approximately 46% lower growth when cultivated with glucose compared to acetate.

### Assessment of glucose uptake ability and efficiency

**Fig. 7:**
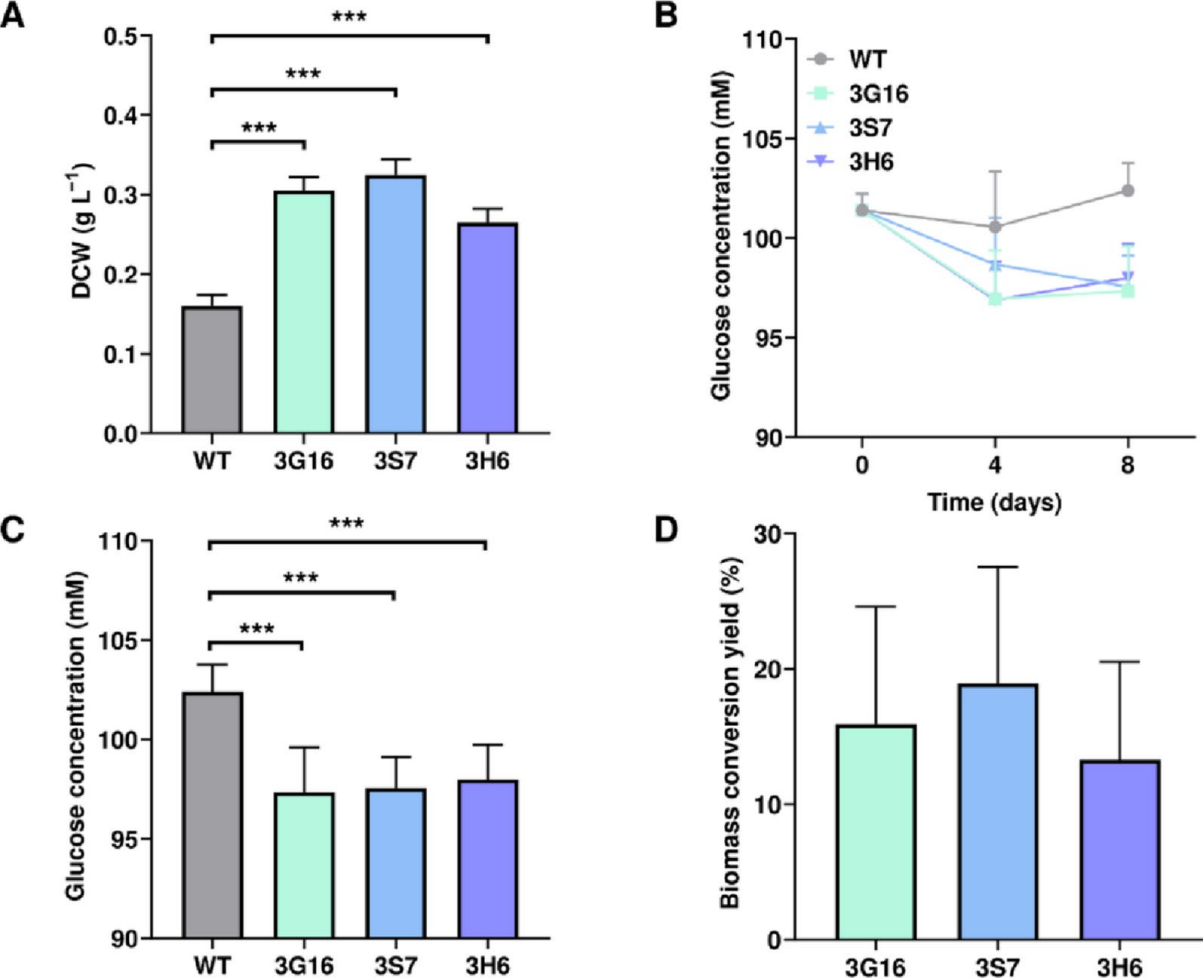
Analyses on the glucose consumption and biomass yield on substrate under dark conditions.**A** DCW of each strain on day 8 under dark conditions in TPG-100 medium. Experiments were conducted in biological duplicates and technical duplicates (*n* = 4). **B** Glucose concentration changes in culture medium measured for 8 days. Experiments were conducted in biological duplicates and technical sextuplicates (*n* = 12 in total). **C** Glucose concentrations in the culture medium on day 8. [***] indicates statistically extremely significant difference (*p* < 0.001) compared to the WT. **D** Glucose-to-biomass conversion yields of each tested strains. Error bar represents the standard deviation of the mean

To assess mutant strains’ actual glucose uptake ability, heterotrophic cultures were initiated at a higher cell density, with the initial OD_800_ set to 0.5, compared to previous dark cultivation experiment was initiated at OD_800_ of 0.1. Glucose uptake was first assessed at 100 mM of glucose to ensure detectable substrate consumption, followed by complementary experiments at lower glucose concentrations (i.e., 5 and 10 mM) to evaluate uptake behavior under more diverse initial glucose conditions. After 8 days of cultivation under TPG-100 conditions, DCW reached 0.16 g L^−1^ in the WT, while mutant strains showed higher values of 0.305, 0.325, and 0.265 g L^−1^ for the 3G16, 3S7, and 3H6 strains, respectively. This corresponded to 1.91-, 2.03-, and 1.66-fold increases compared to the WT (Fig. 7A). Meanwhile, measurements of residual glucose concentration in the culture medium on day 8 revealed final glucose concentrations of 102.39, 97.33, 97.55, and 98.00 mM for the WT, 3G16, 3S7, and 3H6, respectively. The observed increase in glucose concentration in the WT culture medium can be attributed to evaporation during cultivation. Based on this observation, the reduction in glucose concentration in the media of mutant strains was calculated relative to the glucose concentration in the WT culture medium. Residual glucose concentrations in the 3G16, 3S7, and 3H6 strains were 5.05, 4.84, and 4.39 mM lower than that in the WT, respectively (Fig. 7B, C). With DCW and reduced glucose concentration data, glucose-to-biomass conversion yields were calculated for each engineered strain under heterotrophic conditions. Strain 3G16 exhibited a yield of 15.9 ± 8.7%, 3S7 achieved 18.9 ± 8.6%, and 3H6 showed 13.3 ± 7.2%. Although the carbon source used in this study differs from acetate, the yield of the strain 3S7 was comparable to the acetate-to-biomass conversion efficiency of approximately 28% previously reported in *C. reinhardtii* (Fig. 7D; Table 4) [41]. Despite the higher glucose consumption observed in the 3G16 and 3H6 strains, the 3S7 mutant demonstrated the most efficient capacity for glucose utilization.

**Table 4:** DCW, consumed glucose, and conversion yield at the final cultivation time point

Strains	DCW on day 8 (g L^−1^)	Consumed glucose (g L^−1^)	Conversion yield (%)
WT	0.160	N/A	N/A
3G16	0.305	0.911	15.9 ± 8.7
3S7	0.325	0.872	18.9 ± 8.6
3H6	0.265	0.790	13.3 ± 7.2

N/A stands for not applicable

To examine the extent of glucose uptake at different initial glucose concentrations, additional tests were conducted under TPG-5 and TPG-10 conditions. Under TPG-5 conditions, no significant increase in DCW was observed by day 8 (Supplementary Information, Fig. S5A), and residual glucose concentrations did not decrease over the cultivation period (Supplementary Information, Fig. S5B). Instead, a slight increase in measured glucose concentration was detected (Fig S5C), which is likely due to medium evaporation during dark incubation rather than active glucose uptake. These results are consistent with the initial screening experiments performed under dark conditions with 5 mM of glucose (Fig. S2A), where no significant growth differences were observed among the tested transformants. On the contrary, under TPG-10 conditions, detectable biomass accumulation and glucose consumption were observed. On day 8, DCW values reached approximately 0.20, 0.2175, 0.23, and 0.2075 g L^−1^ for the WT, 3G16, 3S7, and 3H6 strains, respectively (Supplementary Information, Fig. S5D). Residual glucose concentrations at the same time point were measured as 10.6, 10.3, 9.9, and 10.3 mM, corresponding to glucose reductions of 0.304, 0.673, and 0.340 mM in the 3G16, 3S7, and 3H6 strains relative to the WT (Supplementary Information, Fig. S5E, F). Based on these values, glucose-to-biomass conversion yields were calculated as 31.9 ± 23.4% for 3G16, 24.8 ± 12.3% for 3S7, and 16.0 ± 20.7% for 3H6 (Supplementary Information, Fig. S5G). However, as the DCW increase of 3H6 and the glucose consumption of 3G16 were not statistically different from those of the WT, we cannot conclude that the derived glucose-to-biomass conversion yields of 3G16 and 3H6 were statistically meaningful. Given that the yields of 3G16, 3S7, and 3H6 were 15.9 ± 8.7%, 18.9 ± 8.6%, and 13.3 ± 7.2%, respectively, at a glucose concentration of 100 mM (Fig. 7), each strain exhibited somewhat higher yields under 10 mM glucose conditions. This inverse relationship between glucose concentration and glucose-to-biomass yield is consistent with a trend previously reported [27].

Through these additional tests, we could obtain several insights into glucose-assisted heterotrophy in *C. reinhardtii*, including the following: (i) A minimum initial glucose concentration likely exists in the range of 5 to 10 mM to induce heterotrophic growth; and (ii) the extent of glucose uptake and the resulting heterotrophic growth appear to depend on the initial external glucose concentration. Of note, the threshold glucose concentration required to initiate heterotrophic growth in *C. reinhardtii* – particularly in the *GLUT1*-exressing strain 3G16 – does not fully align with a previous report [14], which employed the same exogenous glucose transporter and demonstrated that 5 mM of glucose was sufficient to induce modest growth in the dark. This discrepancy may be attributed to several differences between the present and previous studies, including (i) the host strain (*C. reinhardtii* UVM4) and (ii) the vector system, such as the use of different promoters and terminators (pDB124 and pHR13). In addition, locus-dependent effects arising from random genomic integration may also contribute to the observed difference, as these factors can collectively influence transporter expression levels and, consequently, the efficiency of glucose uptake and utilization.

Overall, although the transformants (representatively 3S7) exhibited significantly improved glucose uptake and growth compared to the WT, further strain optimization is still required to achieve industrial applicability under glucose-based heterotrophic cultivation. This could be related to inefficient glucose partitioning within *C. reinhardtii*, as metabolic processing of glucose is known to occur across multiple compartments, potentially limiting the efficiency of carbon flux toward biomass production [42]. Therefore, simple glucose transport into the cytoplasm may not be sufficient due to the missing links within the glycolysis pathway, limiting its entry into the early stages of glycolysis. These challenges highlight the need for further genetic modification of the strain to enhance its ability to fully utilize external glucose. Addressing these issues could draw attention to developing an efficient *C. reinhardtii* strain capable of optimized glucose utilization for industrial applications.

## Conclusions

This study investigated the feasibility of glucose-based heterotrophic cultivation in *C. reinhardtii* through the heterologous expression of glucose transporters, including *HsGLUT1*, *PkHUP1*, and *AtSTP1*. Of the tested strains, 3S7, which functionally expresses *AtSTP1* (a proton/hexose symporter originating from *A. thaliana*) was found to exhibit the highest biomass production and glucose-to-biomass conversion yield under heterotrophic conditions. However, the observed productivity did not reach satisfactory levels. This merits further engineering efforts to define the remaining missing links in glycolysis and to rewire supportive biological pathways, which would confer a more streamlined ability to efficiently utilize glucose upon the model alga. Nevertheless, the present study demonstrates the potential for cultivating *C. reinhardtii* using glucose, the most affordable industrial organic carbon substrate, via the heterologous expression of *AtSTP1*, which could be a viable option for future studies seeking to unlock *C. reinhardtii*’s industrial potential by enabling its heterotrophic, high cell density culture.

## Supplementary Information

Below is the link to the electronic supplementary material.

Supplementary Material 1.

## Data Availability

The data and materials are available from the corresponding author upon request.
