# Supplementary material for: The sugar transporter STP1-driven trophic conversion of Chlamydomonas reinhardtii
Source: Microb Cell Fact. 2026 Feb 20;25:79. doi: 10.1186/s12934-026-02957-4 (PMC13032357; doi:10.1186/s12934-026-02957-4)
Supplement: Supplementary file 1 — Supplementary Material 1. [file 12934_2026_2957_MOESM1_ESM.docx]

| **Table S1.** Codon-optimized gene sequence of glucose transporters | |
| --- | --- |
| Transporter | Codon optimized sequence |
| *HsGLUT1* | ATGGAGCCCAGCAGCAAGAAGCTGACCGGCCGCCTGATGCTGGCTGTCGGCGGCGCTGTGCTGGGCAGCCTGCAGTTCGGCTACAACACCGGCGTGATCAACGCTCCCCAGAAGGTCATCGAGGAGTTCTACAACCAGACCTGGGTGCACCGCTACGGCGAGAGCATTCTGCCCACCACGCTGACGACCCTGTGGTCCCTGTCCGTGGCTATTTTCAGCGTCGGCGGCATGATCGGCAGCTTCAGCGTGGGCCTGTTCGTGAACCGCTTCGGCCGCCGCAACTCCATGCTGATGATGAACCTGCTGGCCTTCGTGTCCGCTGTCCTGATGGGCTTCAGCAAGCTGGGCAAGAGCTTCGAGATGCTGATCCTGGGCCGCTTCATCATCGGCGTGTACTGCGGCCTGACCACCGGCTTCGTGCCGATGTACGTCGGCGAGGTGTCGCCCACCGCTCTGCGCGGCGCTCTGGGCACGCTGCACCAGCTGGGCATTGTCGTGGGCATCCTGATCGCTCAGGTGTTCGGCCTGGACAGCATCATGGGCAACAAGGACCTGTGGCCGCTGCTGCTGAGCATCATCTTCATTCCCGCTCTGCTGCAGTGCATCGTGCTGCCGTTCTGCCCCGAGTCGCCCCGCTTCCTGCTGATTAACCGCAACGAGGAGAACCGCGCCAAGAGCGTGCTGAAGAAGCTGCGCGGCACCGCTGACGTGACCCACGACCTGCAGGAGATGAAGGAGGAGTCCCGCCAGATGATGCGCGAGAAGAAGGTCACCATCCTGGAGCTGTTCCGCTCGCCGGCTTACCGCCAGCCGATTCTGATTGCTGTGGTGCTGCAGCTGAGCCAGCAGCTGTCCGGCATTAACGCCGTGTTCTACTACAGCACCAGCATCTTCGAGAAGGCCGGCGTGCAGCAGCCCGTGTACGCTACCATTGGCAGCGGCATTGTGAACACCGCCTTCACCGTGGTGTCGCTGTTCGTGGTCGAGCGCGCTGGCCGGCGCACCCTGCACCTGATTGGCCTGGCTGGCATGGCTGGCTGCGCTATCCTGATGACCATTGCTCTGGCTCTGCTGGAGCAGCTGCCCTGGATGAGCTACCTGAGCATCGTGGCCATCTTCGGCTTCGTCGCCTTCTTCGAGGTCGGCCCCGGCCCGATTCCGTGGTTCATTGTGGCTGAGCTGTTCTCGCAGGGCCCGCGCCCGGCTGCTATTGCTGTCGCTGGCTTCTCCAACTGGACCAGCAACTTCATCGTCGGCATGTGCTTCCAGTACGTCGAGCAGCTGTGCGGCCCCTACGTGTTCATCATTTTCACCGTGCTGCTGGTCCTGTTCTTCATCTTCACCTACTTCAAGGTGCCCGAGACCAAGGGCCGCACCTTCGACGAGATTGCTTCGGGCTTCCGCCAGGGCGGCGCTAGCCAGAGCGACAAGACGCCCGAGGAGCTGTTCCACCCGCTGGGCGCTGACAGCCAGGTG |
| *AtSTP1* | ATGCCCGCTGGCGGCTTCGTCGTCGGCGACGGCCAGAAGGCTTACCCCGGCAAGCTGACCCCGTTCGTGCTGTTCACGTGCGTGGTGGCTGCTATGGGCGGCCTGATTTTCGGCTACGACATCGGCATTAGCGGCGGCGTGACCTCGATGCCCAGCTTCCTGAAGCGGTTCTTCCCCAGCGTGTACCGCAAGCAGCAGGAGGACGCTAGCACCAACCAGTACTGCCAGTACGACAGCCCCACGCTGACCATGTTCACCAGCAGCCTGTACCTGGCCGCTCTGATTAGCAGCCTGGTGGCTAGCACCGTGACGCGCAAGTTCGGCCGCCGCCTGAGCATGCTGTTCGGCGGCATTCTGTTCTGCGCTGGCGCCCTGATTAACGGCTTCGCTAAGCACGTGTGGATGCTGATCGTGGGCCGCATTCTGCTGGGCTTCGGCATTGGCTTCGCCAACCAGGCTGTGCCGCTGTACCTGAGCGAGATGGCTCCCTACAAGTACCGCGGCGCTCTGAACATCGGCTTCCAGCTGAGCATCACCATCGGCATCCTGGTCGCTGAGGTGCTGAACTACTTCTTCGCCAAGATCAAGGGCGGCTGGGGCTGGCGCCTGAGCCTGGGCGGCGCTGTGGTGCCCGCGCTGATTATTACCATTGGCTCCCTGGTGCTGCCCGACACGCCCAACTCCATGATTGAGCGCGGCCAGCACGAGGAGGCTAAGACCAAGCTGCGCCGCATTCGCGGCGTGGACGACGTGTCCCAGGAGTTCGACGACCTGGTCGCCGCTAGCAAGGAGAGCCAGAGCATTGAGCACCCCTGGCGCAACCTGCTGCGCCGGAAGTACCGGCCGCACCTGACGATGGCTGTGATGATCCCGTTCTTCCAGCAGCTGACCGGCATCAACGTGATCATGTTCTACGCCCCGGTCCTGTTCAACACGATCGGCTTCACCACCGACGCCAGCCTGATGAGCGCTGTCGTGACCGGCAGCGTGAACGTGGCGGCTACCCTGGTGTCCATCTACGGCGTCGACCGCTGGGGCCGCCGCTTCCTGTTCCTGGAGGGCGGCACCCAGATGCTGATTTGCCAGGCTGTCGTGGCCGCTTGCATCGGCGCTAAGTTCGGCGTCGACGGCACCCCGGGCGAGCTGCCCAAGTGGTACGCTATTGTGGTGGTCACCTTCATCTGCATCTACGTGGCGGGCTTCGCTTGGAGCTGGGGCCCGCTGGGCTGGCTGGTGCCCAGCGAGATTTTCCCGCTGGAGATTCGCTCCGCCGCTCAGAGCATTACCGTGTCCGTCAACATGATCTTCACCTTCATTATCGCCCAGATTTTCCTGACCATGCTGTGCCACCTGAAGTTCGGCCTGTTCCTGGTGTTCGCCTTCTTCGTGGTGGTCATGTCGATCTTCGTGTACATCTTCCTGCCGGAGACCAAGGGCATCCCCATTGAGGAGATGGGCCAGGTCTGGCGCAGCCACTGGTACTGGTCCCGCTTCGTCGAGGACGGCGAGTACGGCAACGCTCTGGAGATGGGCAAGAACTCGAACCAGGCTGGCACCAAGCACGTG |
| *PkHUP1* | ATGGCTGGCGGCGGCGTGGTGGTCGTGTCCGGCCGGGGCCTGAGCACCGGCGACTACCGCGGCGGCCTGACCGTGTACGTGGTCATGGTGGCTTTCATGGCCGCTTGCGGCGGCCTGCTGCTGGGCTACGACAACGGCGTGACCGGCGGCGTCGTGTCCCTGGAGGCTTTCGAGAAGAAGTTCTTCCCCGACGTCTGGGCCAAGAAGCAGGAGGTCCACGAGGACAGCCCCTACTGCACGTACGACAACGCTAAGCTGCAGCTGTTCGTGTCCAGCCTGTTCCTGGCTGGCCTGGTGTCGTGCCTGTTCGCTAGCTGGATTACCCGCAACTGGGGCCGCAAGGTCACCATGGGCATTGGCGGCGCTTTCTTCGTCGCTGGCGGCCTGGTCAACGCTTTCGCTCAGGACATGGCTATGCTGATCGTGGGCCGCGTCCTGCTGGGCTTCGGCGTCGGCCTGGGCAGCCAGGTGGTGCCCCAGTACCTGAGCGAGGTGGCGCCCTTCAGCCACCGCGGCATGCTGAACATTGGCTACCAGCTGTTCGTCACCATCGGCATCCTGATCGCTGGCCTGGTCAACTACGCTGTGCGCGACTGGGAGAACGGCTGGCGCCTGTCGCTGGGCCCCGCCGCTGCTCCCGGCGCTATTCTGTTCCTGGGCTCCCTGGTGCTGCCCGAGTCGCCCAACTTCCTGGTCGAGAAGGGCAAGACCGAGAAGGGCCGCGAGGTGCTGCAGAAGCTGTGCGGCACCTCCGAGGTGGACGCTGAGTTCGCTGACATTGTGGCCGCCGTCGAGATTGCTCGCCCCATTACCATGCGCCAGAGCTGGGCTAGCCTGTTCACCCGCCGCTACATGCCCCAGCTGCTGACCAGCTTCGTGATCCAGTTCTTCCAGCAGTTCACCGGCATCAACGCCATCATCTTCTACGTGCCCGTGCTGTTCAGCTCGCTGGGCAGCGCTAACAGCGCTGCTCTGCTGAACACCGTGGTCGTGGGCGCTGTGAACGTGGGCTCGACCCTGATTGCTGTGATGTTCAGCGACAAGTTCGGCCGCCGGTTCCTGCTGATTGAGGGCGGCATTCAGTGCTGCCTGGCCATGCTGACCACCGGCGTGGTCCTGGCTATCGAGTTCGCTAAGTACGGCACGGACCCGCTGCCGAAGGCTGTGGCTAGCGGCATTCTGGCCGTGATCTGCATCTTCATCAGCGGCTTCGCTTGGAGCTGGGGCCCGATGGGCTGGCTGATTCCCAGCGAGATTTTCACCCTGGAGACGCGCCCCGCTGGCACCGCTGTGGCTGTCGTGGGCAACTTCCTGTTCTCGTTCGTGATCGGCCAGGCCTTCGTGTCGATGCTGTGCGCTATGGAGTACGGCGTGTTCCTGTTCTTCGCTGGCTGGCTGGTCATCATGGTGCTGTGCGCCATTTTCCTGCTGCCGGAGACCAAGGGCGTGCCCATTGAGCGCGTGCAGGCTCTGTACGCTCGCCACTGGTTCTGGAACCGCGTGATGGGCCCCGCTGCCGCTGAGGTGATCGCTGAGGACGAGAAGCGCGTGGCCGCTGCTAGCGCCATTATTAAGGAGGAGGAGCTGAGCAAGGCCATGAAG |


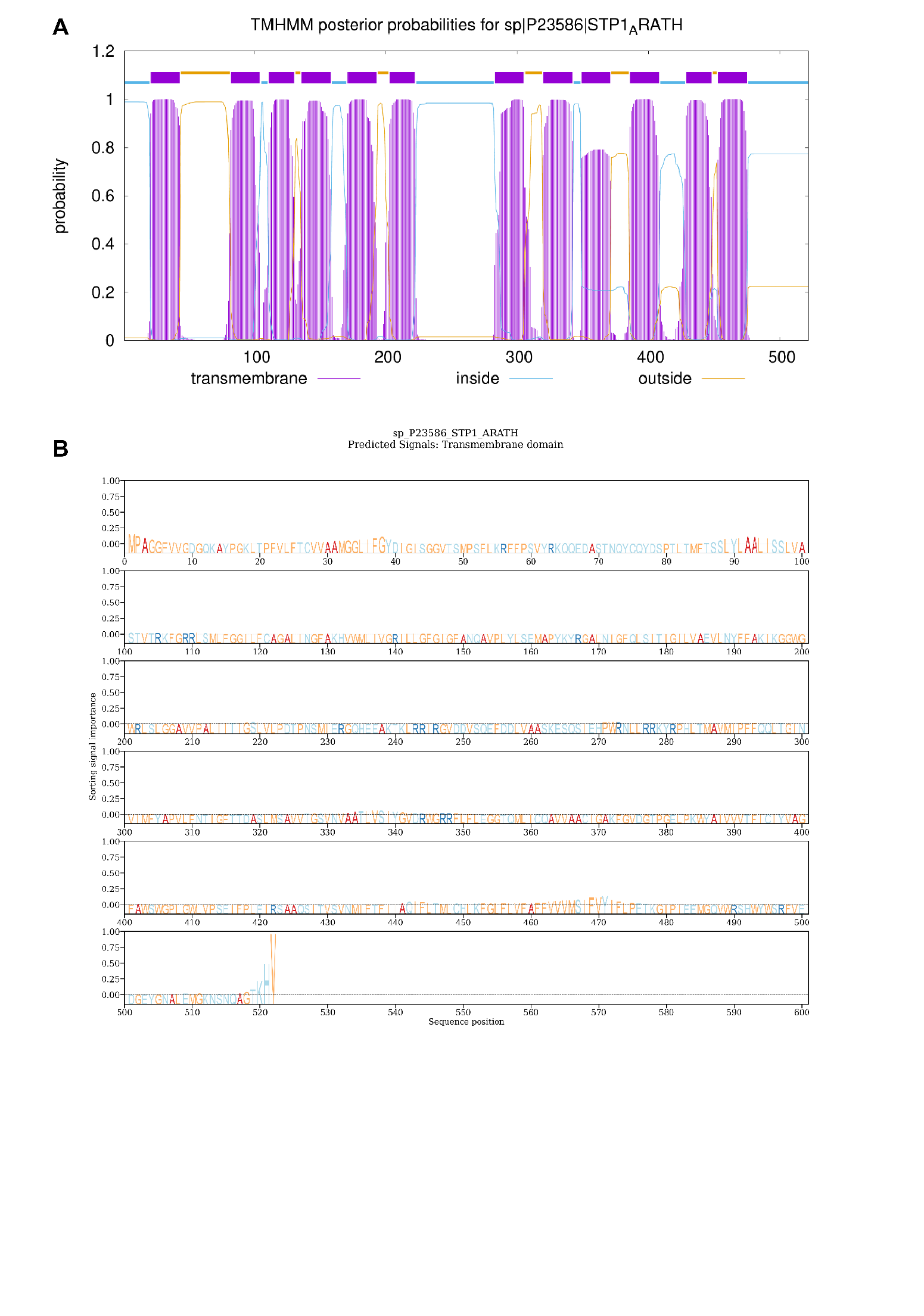


**Fig. S1. Graphical results of *in silico* analyses predicting transmembrane topology and subcellular localization of STP1.** (A) Prediction result from TMHMM v2.0 suggested that the STP1 protein may possess 12 transmembrane helices. (B) DeepLoc v2.1 analysis showed that STP1 is predicted to be a membrane-associated protein, implying its localization.


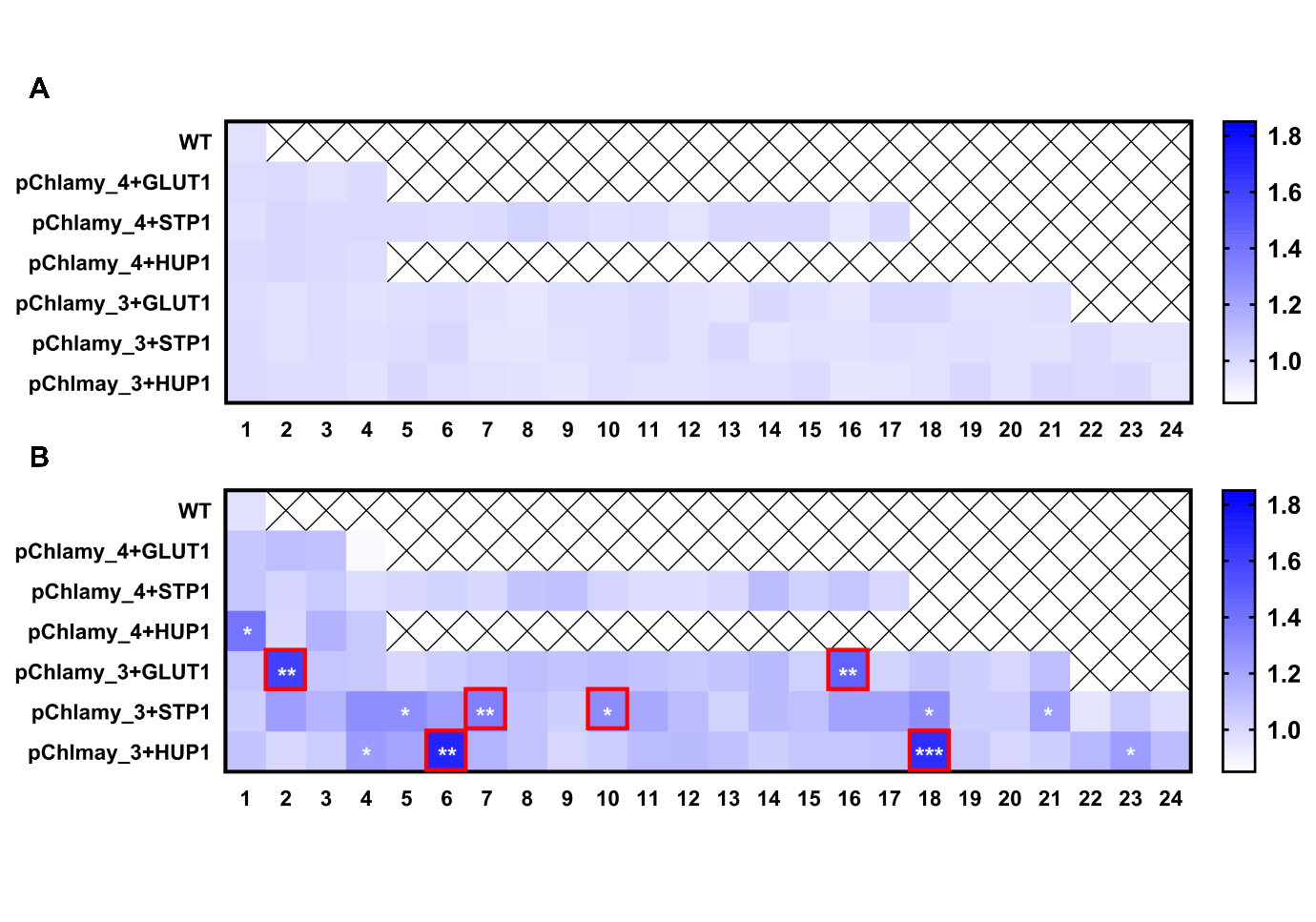
**Fig. S2. Initial screening of *C. reinhardtii* transformants expressing heterologous glucose transporters.** Transformants harboring each plasmid were cultivated under (A) dark conditions and (B) light conditions in TPG-5 medium for 3 days. The heatmap shows the fold change in growth, calculated by dividing the OD_800_ value at day 3 by that at day 0. Each square represents an individual transformant, and color intensity indicates relative growth. Transformants highlighted in red were selected as primary candidates during the initial screening step and subjected to subsequent characterization (Fig. S3). Experiments were conducted with technical quadruplicates (n = 4). [*], [**], and [***] indicate a significant difference (*p* < 0.05), a highly significant difference (*p* < 0.01), and an extremely significant difference (*p* < 0.001), respectively, compared to the wild-type (WT).

**
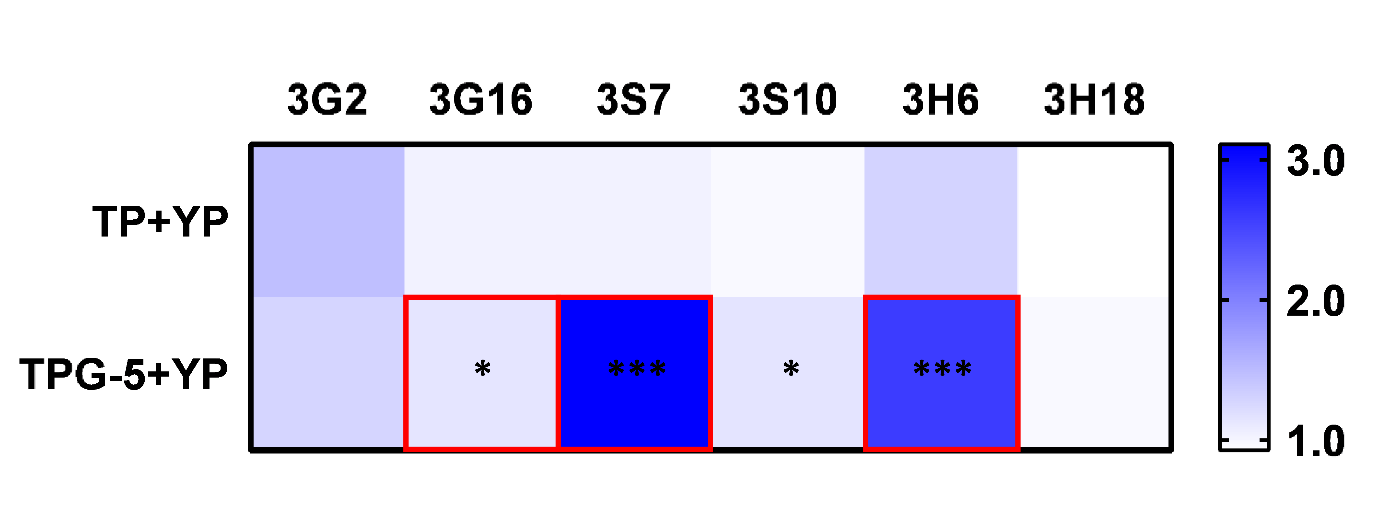
Fig. S3. Growth evaluation of selected mutant strains harboring the pChlamy_3-based vector under nutrient-enriched conditions.** Candidate transformants were previously selected from the growth test in 12-well plates (Fig. S2). From each transporter-harboring group, two strains exhibiting the highest growth were selected and further evaluated in complex media conditions. Strains were cultivated in 24-well plates for 3 days, and OD₈₀₀ was measured at day 0 and 3. Growth fold change was calculated as the ratio of OD₈₀₀ at day 3 to that at day 0 and visualized as a heatmap. Experiments were conducted with biological triplicates and technical quadruplicates (n = 12 in total). [*] and [***] indicate a significant difference (*p* < 0.05) and an extremely significant difference (*p* < 0.001), respectively, compared to the TP+YP results of each strain. Selected transformants for further evaluation are highlighted in red.


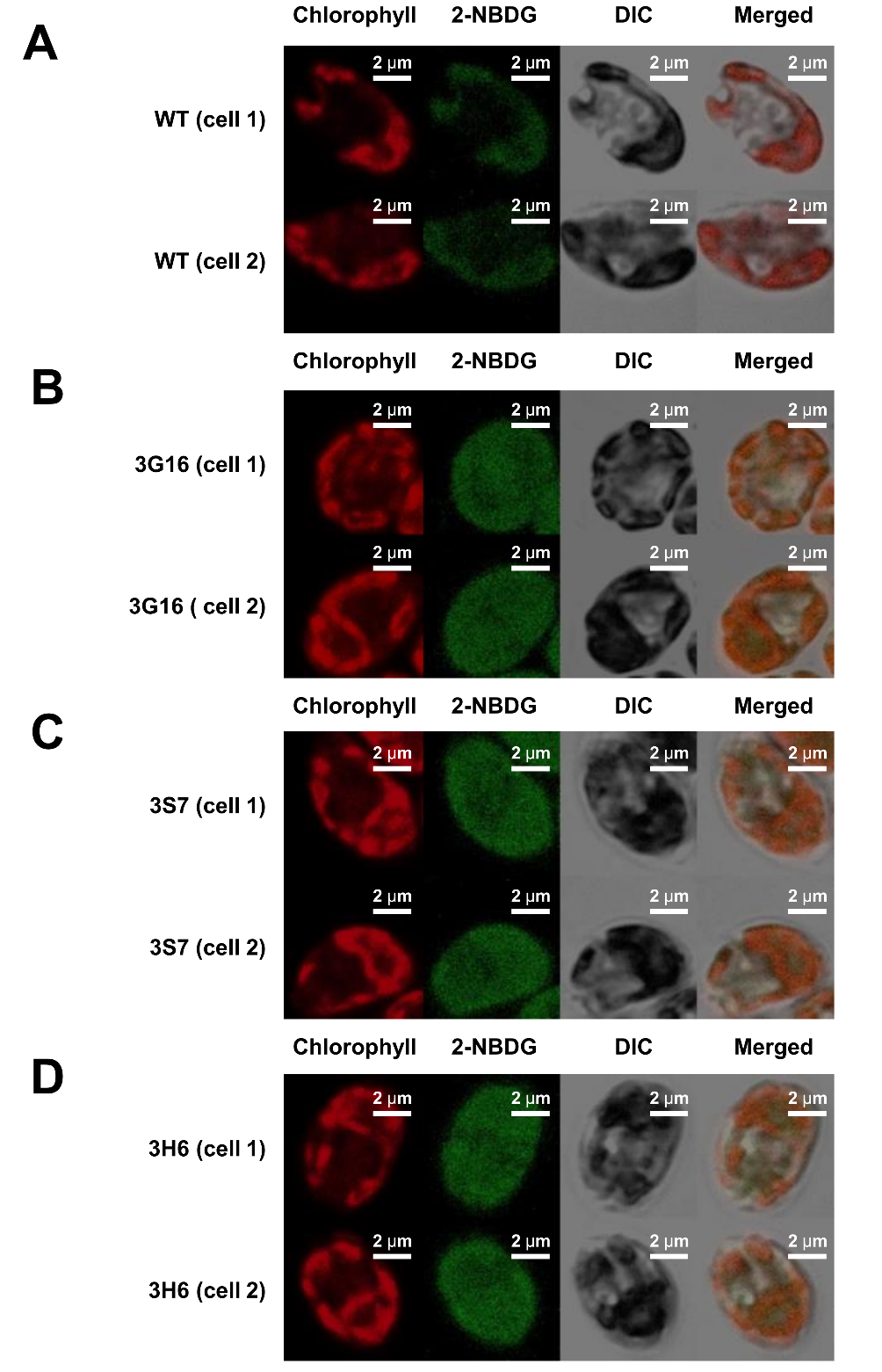


**Fig. S4. Representative confocal images used for ImageJ-based quantification of 2-NBDG fluorescence quantification.** Representative single-cell images of (A) WT, (B) 3G16, (C) 3S7, and (D) 3H6 strains are shown, with two individual cells displayed per strain. Images were acquired using the same confocal imaging settings as those applied in Fig. 3B. For quantitative analysis, ROIs were manually defined for each individual cell. Chlorophyll, 2-NBDG, differential interference contrast (DIC), and merged channels are depicted.

*
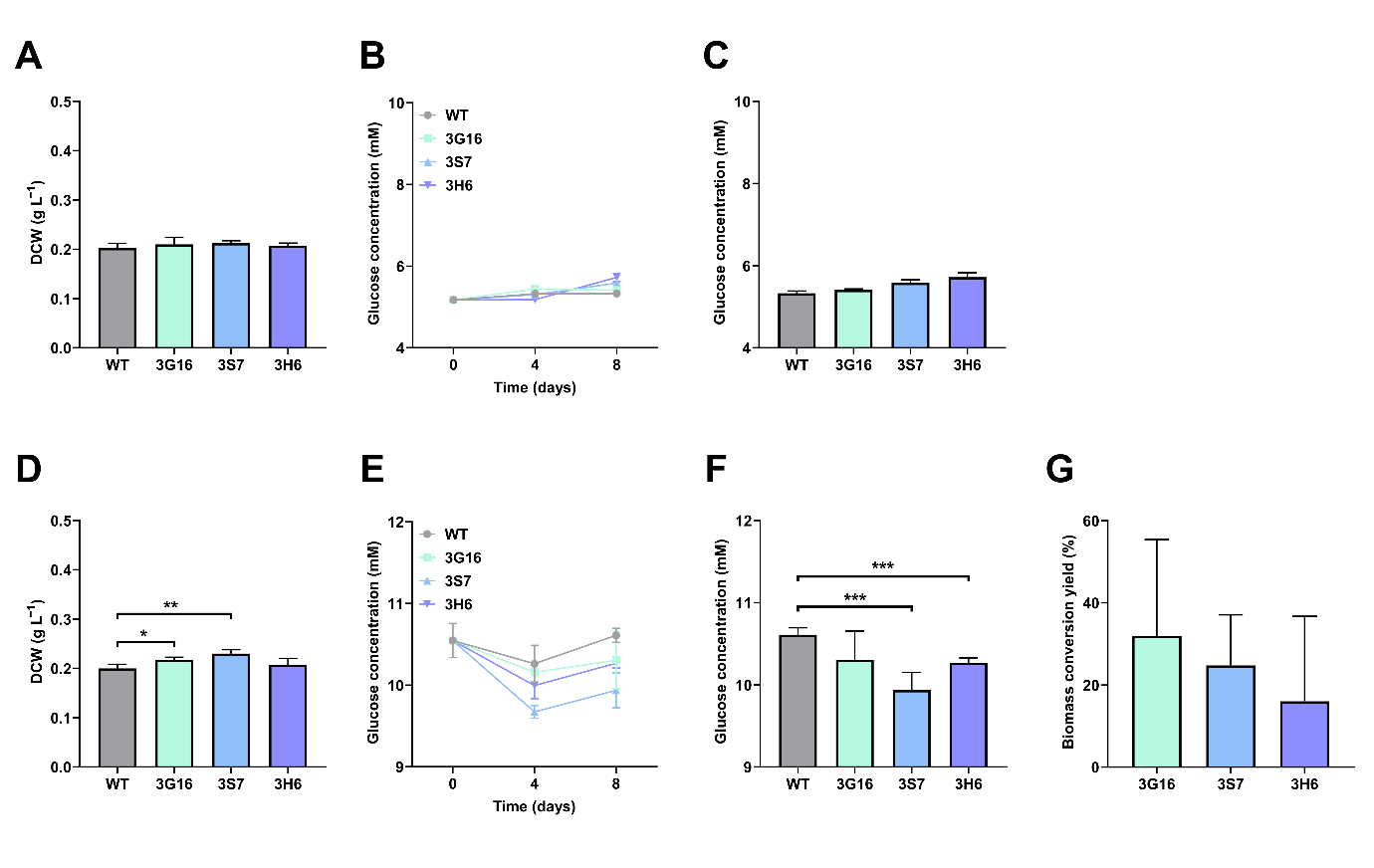
*

**Fig. S5.** **Analyses of glucose consumption and biomass yield on substrate under dark conditions at low initial glucose concentrations.** The WT and glucose transporter-expressing strains were cultivated under heterotrophic conditions using TPG-5 (A–C) and TPG-10 media (D–G). (A) DCW measured at day 8 under TPG-5 conditions. (B) Residual glucose concentration in the culture medium measured at days 0, 4, and 8 under TPG-5 conditions. (C) Residual glucose concentration in the culture medium at day 8 under TPG-5 conditions. (D) DCW measured at day 8 under TPG-10 conditions. (E) Residual glucose concentration in the culture medium measured at days 0, 4, and 8 under TPG-10 conditions. (F) Residual glucose concentration in the culture medium at day 8 under TPG-10 conditions. (G) Biomass conversion yield (%) at day 8 under TPG-10 conditions. DCW measurements in panels A and D were conducted with biological duplicates and technical duplicates (n = 4 in total), and residual glucose concentrations in panels B, C, E, and F were measured with biological duplicates and technical triplicates (n = 6 in total). [***] indicates statistically extremely significant difference (*p* < 0.001) compared to the WT. Error bars represent the standard deviation of the mean.
